# Gluten‐Free Diet Induces Small‐Scale Changes Across Multiple T‐Cell Subsets in NOD Mice

**DOI:** 10.1002/eji.202451559

**Published:** 2025-04-21

**Authors:** Veronika Niederlova, Juraj Michalik, Barbora Drabonova, Radka Cisarova, David Funda, Ondrej Stepanek

**Affiliations:** ^1^ Laboratory of Adaptive Immunity Institute of Molecular Genetics of the Czech Academy of Sciences Prague Czech Republic; ^2^ Laboratory of Cellular and Molecular Immunology Institute of Microbiology v.v.i., Czech Academy of Sciences Prague Czech Republic; ^3^ Department of Microbiology, Nutrition and Dietetics, Faculty of Agrobiology, Food and Natural Resources Czech University of Life Sciences Prague Prague Czech Republic

**Keywords:** type I diabetes, T regulatory cells, gluten‐free diet, single‐cell transcriptomics, NOD mice

## Abstract

Nonobese diabetic (NOD) mice are a widely used animal model to study mechanisms leading to autoimmune diabetes. A gluten‐free diet reduces and delays the incidence of diabetes in NOD mice, but the underlying mechanisms remain largely unknown. In this study, we performed single‐cell transcriptomic and flow cytometry analysis of T cells and innate lymphocytes in the spleen and pancreatic lymph nodes of NOD mice fed a gluten‐free or standard diet. We observed that the gluten‐free diet did not induce a substantial alteration in the abundance or phenotype of any lymphocyte subset that would directly explain its protective effect against diabetes. However, the gluten‐free diet induced subtle changes in the differentiation of subsets with previously proposed protective roles in diabetes development, such as Tregs, activated γδT cells, and NKT cells. Globally, the gluten‐free diet paradoxically promoted activation and effector differentiation across multiple subpopulations and induced genes regulated by IL‐2, IL‐7, and IL‐15. In contrast, the standard diet induced type I interferon‐responsive genes. Overall, the gluten‐free diet might prevent diabetes in NOD mice by inducing small‐scale changes in multiple cell types rather than acting on a specific lymphocyte subset.

## Graphical abstract

A gluten‐free diet prevents Type I diabetes in NOD mice. We characterized T cells from prediabetic mice on gluten‐free or standard diets using flow cytometry and single‐cell transcriptomics. A gluten‐free diet enhanced T‐cell activation and effector differentiation, including regulatory T cells, suggesting immune modulation as a mechanism for diabetes prevention.

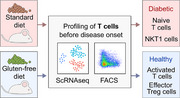

**Abbreviations**
AIMT: antigen inexperienced memory‐like cells
DEG: differentially expressed genes
FACS: fluorescence‐activated cell sorting
FC: flow cytometry
GFD: gluten‐free diet
GSEA: gene set enrichment
IFN: interferon
ILC: innate lymphoid cells
iLN: inguinal lymph nodes
IREA: immune response enrichment analysis
NKT: natural killer T cells
NOD: nonobese diabetic
pLN: pancreatic lymph nodes
SCID: severe combined immunodeficiency
scRNAseq: single‐cell RNA sequencing
STD: standard diet
T1D: type I diabetes
TCR: T‐cell receptor
Tfh: follicular helper T cells
Treg: regulatory T cells
UMAP: uniform manifold approximation and projection
γδT: γδ T cells

## 1 Introduction

Type 1 diabetes mellitus (T1D) is a severe autoimmune disease affecting more than 8.4 million people worldwide [1]. Nonobese diabetic (NOD) mouse is an inbred strain with a high incidence of spontaneous T1D, for which it is used as the most common animal model of T1D [2]. NOD mice spontaneously develop the disease and recapitulate some key aspects of the human disease, including a strong genetic component of a particular MHC‐II allele, I‐Ab(g7) [3, 4, 5]. In both mice and humans, the pathogenesis is mediated by self‐reactive T cells targeting insulin‐producing β cells in the islets of the pancreas [6, 7]. On the other hand, regulatory T cells (Tregs) suppress the function of self‐reactive effector cells and can thus prevent the disease or ameliorate its manifestation [8].

It is well established that environmental factors play a role in the development of T1D based on the twin studies [9, 10] and the global increase of T1D incidence in the past decades [11]. However, these factors are largely elusive. NOD mice represent a useful model for studying the role of various environmental factors in T1D development in controlled experiments. One such notable example is the role of microbiota as revealed by higher incidence and accelerated onset of T1D in germ‐free NOD mice compared with NOD mice housed in specific pathogen‐free facilities [12, 13, 14]. Moreover, it has been observed in multiple studies that a gluten‐free diet (GFD) has a protective role in NOD mice [15, 16, 17, 18, 19, 20]. A promising partially protective effect of GFD was shown in a human study, although a larger cohort would be required for definitive conclusions [21]. However, the molecular and cellular mechanisms of how gluten increases T1D susceptibility are largely unclear.

Recently, single‐cell RNA sequencing (scRNAseq) revolutionized the field of immunology, allowing the characterization of cell types and states in an unprecedented manner. Accordingly, scRNAseq has been successfully used to study distinct aspects of disease development in NOD mice [22, 23, 24, 25], including a recent study focusing on the effect of different diets on T1D development [26]. While these studies significantly enhanced our understanding of immune populations potentially involved in disease progression and regulation, it is still unclear how particular immune subsets respond to environmental stimuli.

In this study, we performed a comprehensive profiling of gene expression and T‐cell receptor (TCR) repertoires of T cells from mice housed on standard diet (STD) or GFD to observe whether these diets differentially regulate the differentiation of T cells and their gene expression.

## 2 Results

**FIGURE 1:**
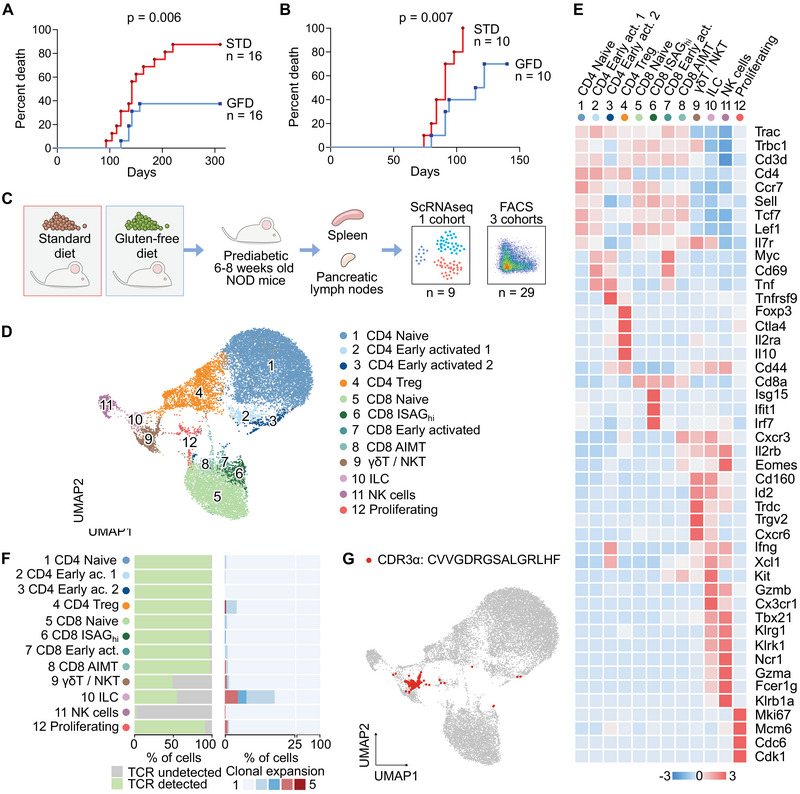
ScRNAseq analysis of mice fed with the GFD or STD. (A) The incidence of diabetes was measured in a cohort of female NOD mice fed with a standard diet containing gluten (*n* = 16) and NOD mice fed with a gluten‐free diet with the same content of total protein, fat, fiber, minerals, and vitamins (*n* = 16). The diagnosis of diabetes was based on two consecutive blood glucose readings >12 mM within three days, the first reading was used as the date of diabetes onset. *p*‐value was calculated with the Log‐rank (Mantel‐Cox) Test. Data from a single experiment. (B) Diabetogenic splenocytes (5 × 10^6^ live cells per mouse) were isolated from 12‐week‐old prediabetic NOD females housed on GFD (*n* = 10) or STD (*n* = 10) and administered intraperitoneally to NOD‐SCID recipients (*n* = 20). The diagnosis of diabetes in host NOD‐SCID mice was based on two consecutive blood glucose readings >12 mM within three days, the first reading was used as the date of diabetes onset. *p*‐value was calculated with the Log‐rank (Mantel‐Cox) Test. Data from a single experiment. (C) Schematic illustration of experimental design of the scRNAseq and flow cytometry analysis. (D–G) Lymphoid populations from spleens and pancreatic lymph nodes of 6‐week‐old NOD mice housed on GFD (*n* = 5) or STD (*n* = 4) were FACS‐sorted and processed by scRNAseq. Data from a single experiment. (D) UMAP projection of 16 818 cells that passed quality control. Cells are colored by manual annotations. (E) Heatmap of marker genes that characterize clusters presented in (D). Color represents the row‐scaled *z*‐score of the average expression of a gene in a cluster. (F) Analysis of gene segments from the TCR locus by the VDJ profiling assay of the scRNAseq. For each cluster, the percentage of cells with detected VDJ transcript (left) and the percentage of clonally expanded cells, that is, cells that shared the same CDR3 TCRβ sequence (right), is shown. (G) The same UMAP projection as in (D). Highlighted are cells with detected NKT‐specific CDR3a sequence CVVGDRGSALGRLHF. AIMT—antigen‐inexperienced memory‐like T cells, CDR—complementarity determining region, FACS—fluorescence activated cell sorting, GFD—gluten‐free diet, ILC—innate lymphoid cells, ISAG—interferon‐stimulated activation genes, NKT—natural killer cells, scRNAseq—single‐cell RNA sequencing, STD—standard diet, TCR—T‐cell receptor, Treg—regulatory T cells, UMAP—uniform manifold approximation and projection.

In this work, we aimed to characterize the peripheral immune populations in mice fed with a gluten‐containing STD or gluten‐deficient GFD to see how these changes contribute to the disease pathology. In our cohort, the disease developed in 87.5% of animals on the STD and only in 37.5% of animals on the GFD (Figure 1A), consistent with previous reports [16, 20, 27]. We reproduced a previous observation [17] that the effect of the diets is maintained even upon the adoptive transfer of splenocytes from animals housed on the STD or GFD to immunodeficient NOD‐SCID mice kept on the STD. The recipients of splenocytes from STD‐fed donors exhibited a higher disease incidence, indicating that the diets control the T1D incidence via direct or indirect effects on the lymphocyte compartment (Figure 1B).

To evaluate the diet‐induced changes before the onset of the disease, we analyzed spleens and pancreatic lymph nodes (pLN) at six weeks of age by scRNAseq and flow cytometry (FC) (Figure 1C). At this early stage, we did not observe any alterations in the frequencies of B cells, T cells, and major T‐cell subsets in the spleen and pLNs (Fig. S1A‐D). In the next step, we characterized the gene expression and TCR repertoire profiles of sorted non‐B‐cell lymphocytes using scRNAseq (Fig. S1E).

### 2.1 ScRNAseq Analysis Reveals Heterogeneity of Cell Populations in pLN and Spleen

After filtering out low‐quality and contaminating cells (Figure S2A–D), we obtained high‐quality transcriptomes of 16,818 cells in total (1771–1923 cells per mouse) (Figure S2E). Unsupervised clustering revealed 19 clusters (Figure S2F). We combined some of the small clusters to obtain twelve clusters in total (Figure 1D), corresponding to canonical populations characterized by established marker genes (Figure 1E; Table S1). Specifically, clusters of conventional T cells were characterized by the expression of TCRαβ (*Trac, Trbc1*) and CD3 (*Cd3d*). These clusters included naïve CD4^+^ and CD8^+^ T cells (*Ccr7, Sell, Tcf7, Lef1* and either *Cd4* or *Cd8a*), early activated CD4^+^ and CD8^+^ T cells (*Myc, Cd69*), CD4^+^ Treg cells (*Foxp3, Ctla4, Il2ra, Il10*), CD8^+^ Antigen‐inexperienced memory‐like cells (AIMT) (*Cd44, Cxcr3, Eomes, Il2rb, Itga4*) [28] and CD8^+^ ISAG^hi^ cells [29, 30]. Unconventional populations included γδ T cells (γδT) (*Trdc, Trgv2, Il7r, Cd160*) and innate lymphoid cells (ILC) (*Kit, Xcl1, Cx3cr1, Id2*). NK cells were characterized by the lack of TCR sequences and NK‐cell markers (*Fcer1g, Klrg1, Klrb1a, Ncr1*). Proliferating cells included distinct cell types that shared expression of cell cycle genes (*Mki67, Mcm6, Cdc6, Cdk1*). The transcriptomic analysis was complemented with V(D)J profiling of TCRα and β sequences. As expected, the TCRαβ sequences were not detected in NK cells (Figure 1F). However, we detected rearranged TCRαβ transcripts in a few cells within the γδT/NKT and ILC clusters, in particular, a conserved CDR3α sequence typical for semi‐invariant NKT cells [31] (Figure 1G).

A comparison of the pLNs and spleens (Figure S3A,B) revealed a significant enrichment of naïve CD4^+^ T cells and early activated CD4^+^ populations in pLNs, while CD4^+^ Treg, γδT/NKT, CD8^+^ AIMT, and proliferating cells were enriched in the spleen. NK cells and ILCs were almost exclusively present only in the spleen (Figure S3B).

We validated our scRNAseq results using an FC analysis of the pLN, spleen, and inguinal lymph nodes (iLN) from the same mice, using a panel of antibodies to characterize the main T‐cell subpopulations. Our unsupervised analysis identified five main populations: two clusters of CD8^+^ T cells, two clusters of CD4^+^ T cells, NK cells, and NKT/γδT cells (Figure S3C). Based on the markers of these subsets (Figure S3D), we merged the scRNAseq clusters to obtain six superclusters that corresponded to those identified in the FC analysis (Figure S3E). The frequencies of these populations in the scRNAseq and FC analyses correlated well (Figure S3F). Accordingly, the frequency of the FC clusters in tissues corresponded to the scRNAseq data (Figures S3B,G). Overall, we obtained high‐resolution scRNAseq data, which were essentially validated using unsupervised FC analysis.

### 2.2 Gluten‐Free Diet Does Not Impair Activation of Conventional T Cells

**FIGURE 2:**
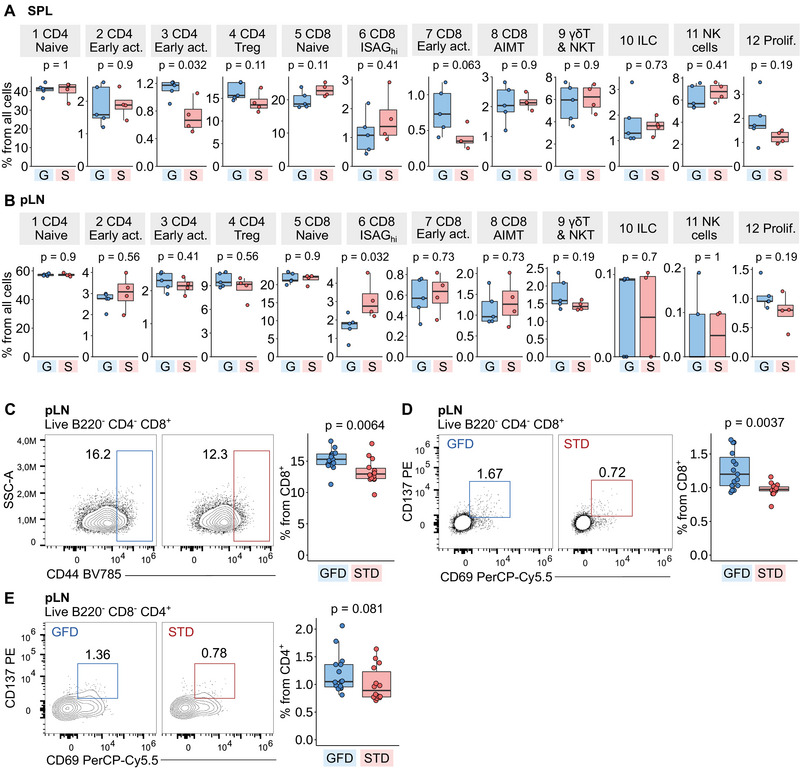
ScRNAseq and flow cytometry analysis of cell subsets in the GFD and STD‐fed mice. (A, B) Quantification of abundance of cell clusters from scRNAseq data shown in Figure 1D in mice housed on a gluten‐free diet (G, blue) or standard diet (S, red). Plotted are percentages of cells in the specified cluster from all cells in the spleen (A) or pancreatic lymph nodes (B). *p*‐values were determined by the two‐tailed Mann–Whitney test. In the boxplots, the line represents the median, the hinges correspond to the first and third quartiles, and the whiskers represent 1.5× interquartile range. *n* = 9 mice from one scRNAseq experiment. (C–E) Flow cytometry analysis of selected cell subsets: CD8^+^ CD44^+^ cells (C), CD69^+^CD137^+^ CD8^+^ T cells (D), and CD69^+^CD137^+^ CD4^+^ T cells (E) in the pLN of mice fed with gluten‐free (GFD, blue) or standard (STD, red) diet. Left—representative gating of the population in pLN sample from one mouse housed on a gluten‐free diet (GFD, blue) and one mouse housed on a standard diet (STD, red). Right—quantification of the percentages of selected T‐cell population from parent population in pLN of mice housed on GFD or STD. Gating and other tissues are shown in Figure S3. *p*‐values were determined by the two‐tailed Mann–Whitney test. *n* = 29 mice from three independent experiments. AIMT—antigen‐inexperienced memory‐like T cells, G or GFD—gluten‐free diet, ILC—innate lymphoid cells, ISAG—interferon‐stimulated activation genes, NKT—natural killer cells, pLN—pancreatic lymph node, SPL—spleen, S or STD—standard diet, Treg—regulatory T cells.

We hypothesized that the tolerogenic GFD would reduce the amount of activated CD4^+^ and/or CD8^+^ T cells in NOD mice and increase the frequency of naïve T cells. However, we did not observe higher frequencies of early activated CD8^+^ T cells (cluster 7), activated CD4^+^ T cells (clusters 2 and 3), or proliferating T cells (cluster 12) in the STD‐fed mice (Figure 2A,B). Cluster 3 of activated CD4^+^ T cells was even slightly more abundant in the spleens of mice kept on the GFD (Figure 2A). Accordingly, the FC analysis using three independent cohorts of STD and GFD mice showed that non‐naïve CD44^+^ CD8^+^ T cells, activated CD69^+^ CD137^+^ CD8^+^ T cells, and activated CD69^+^ CD137^+^ CD4^+^ T cells were not reduced, but rather slightly expanded in the GFD‐fed mice in comparison to the STD‐fed mice in the pLNs (Figure 2C–E; Figure S4A–G). These results suggested that the GFD does not impair the formation of activated or generally non‐naïve conventional CD4^+^ and CD8^+^ T cells globally, and thus, it probably acts via different mechanisms.

### 2.3 Differences in the T‐Regulatory Compartment

**FIGURE 3:**
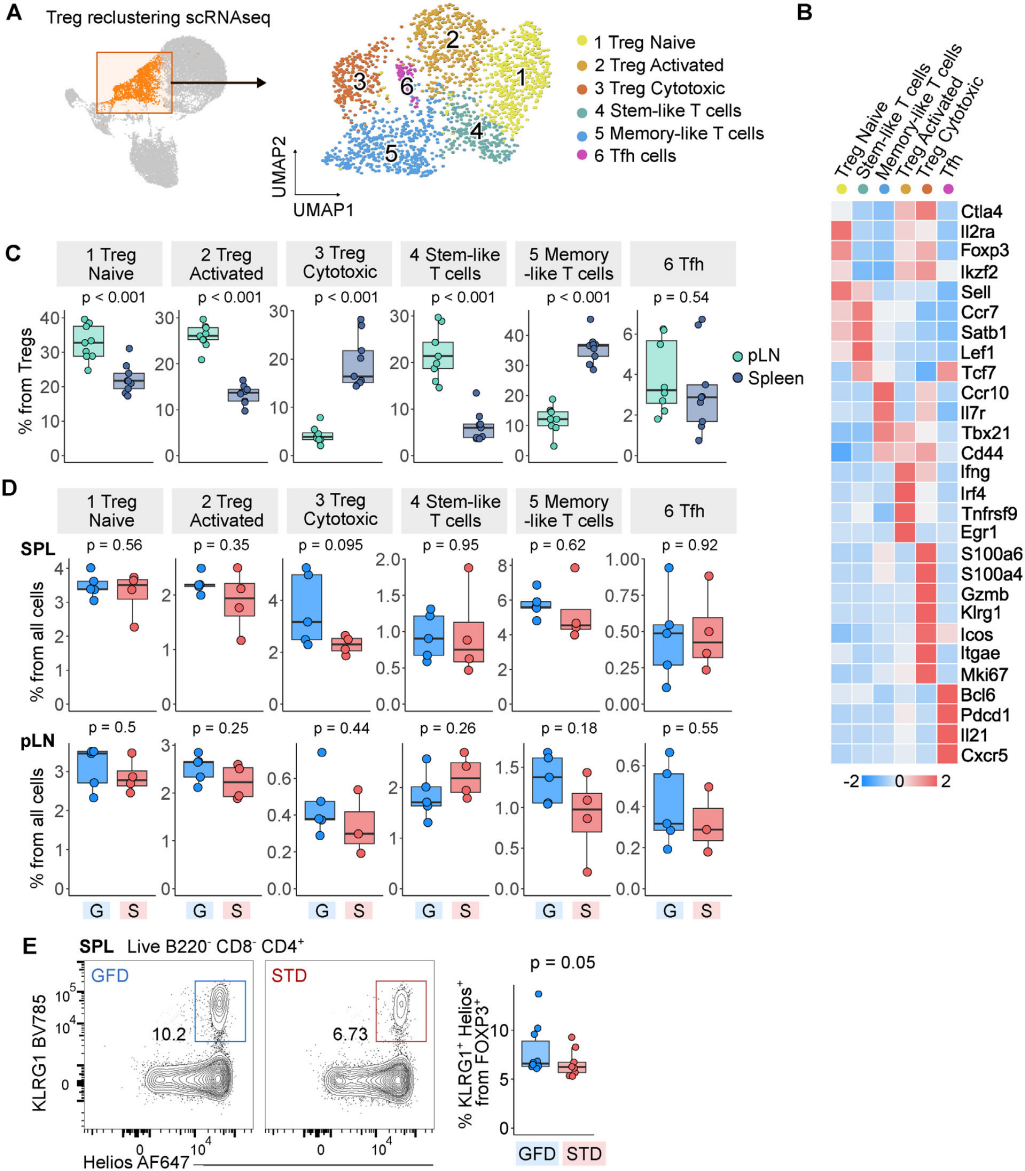
Characterization of Treg cells. (A–D) Reclustering of Treg clusters from the UMAP projection is shown in Figure 1D. Selected clusters were extracted from the dataset and subjected to new normalization, scaling, and dimensional reduction. *n* = 2019 cells from nine mice in one scRNAseq experiment. (A) The UMAP projection from the reclustering of Treg cells showing the clusters used for reclustering (left) and the six new clusters formed (right). Cells are colored by manual annotations. (B) Heatmap of marker genes that characterize clusters presented in (A). Color represents the row‐scaled *z*‐score of the average expression of a gene in a cluster. (C) Quantification of abundance of clusters shown in (A) in the two organs analyzed. Plotted are percentages of cells in the specified cluster from all Tregs. *p*‐values were determined by a two‐tailed Mann‐Whitney test without correction for multiple comparisons. (D) Quantification of abundance of clusters shown in (A) in mice housed on the two different diets. Plotted are percentages of cells in the specified cluster from all cells. *p*‐values were determined by a two‐tailed Mann–Whitney test. S—standard diet, G—gluten‐free diet. (E) Flow cytometry analysis of manually gated KLRG1^+^ Helios^+^ Treg. Left—representative gating of the population in spleen sample from one mouse housed on a gluten‐free diet (GFD, blue) and one mouse housed on a standard diet (STD, red). Right—quantification of the percentages of KLRG1^+^ Helios^+^ Treg from all FOXP3^+^ Tregs in mice on GFD and STD. Full gating and other tissues are shown in Figure S4. *p*‐values were determined by the one‐tailed Mann–Whitney test. *n* = 19 mice from two independent experiments. In the boxplots, the line represents the median, the hinges correspond to the first and third quartiles, and the whiskers represent 1.5× interquartile range. G or GFD—gluten‐free diet, pLN—pancreatic lymph node, SPL—spleen, S or STD—standard diet, Tfh—Follicular helper T cells, Treg—Regulatory T cells.

As it has been proposed that gluten negatively regulates Treg numbers [32], we extracted and re‐analyzed cells from the CD4^+^ Treg cluster, which formed six subpopulations (Figure 3A). Three of these populations were bona fide Tregs as they expressed established Treg markers *Foxp3*, *Il2ra* (CD25), *Ctla4*, and *Ikzf2* (Helios). We annotated these populations as naïve, activated, and cytotoxic Tregs based on their expression profiles (Figure 3A,B). The other three subsets were follicular helper T cells (Tfh), stem‐like, and memory‐like T cells without a clear Treg signature. We observed that the cytotoxic Tregs and memory‐like T cells were more frequent in the spleen than in the pLN, whereas naïve Tregs, activated Tregs, and stem‐like T cells were more abundant in the pLN (Figure 3C). The comparison of the population frequencies in the GFD‐fed and control mice revealed a slight enrichment of cytotoxic Tregs in GFD‐fed mice in the spleen (Figure 3D). In the next step, we used FC to validate this observation. The frequency of cytotoxic Tregs, gated as a KLRG1^+^ Helios^+^ subset among FOXP3^+^ Tregs, was slightly increased in the spleens of GFD‐fed mice (Figure 3E; Figure S5A–C). KLRG1^+^ Tregs had high levels of CD103 and low levels of TCF7, which added further evidence that this subpopulation corresponds to the cytotoxic Tregs observed in the scRNAseq data (Figure 3B; Figure S5D,E). Compared with KLRG1^−^ Tregs, KLRG1^+^ Tregs showed higher proliferation measured by increased Ki‐67 levels (Figure S5F), suggesting an active expansion of this subset. Overall, this analysis suggested that GFD might promote the maturation and proliferation of cytotoxic Tregs.

### 2.4 Gluten‐Free Diet Alters the Phenotype of Unconventional T Cells

**FIGURE 4:**
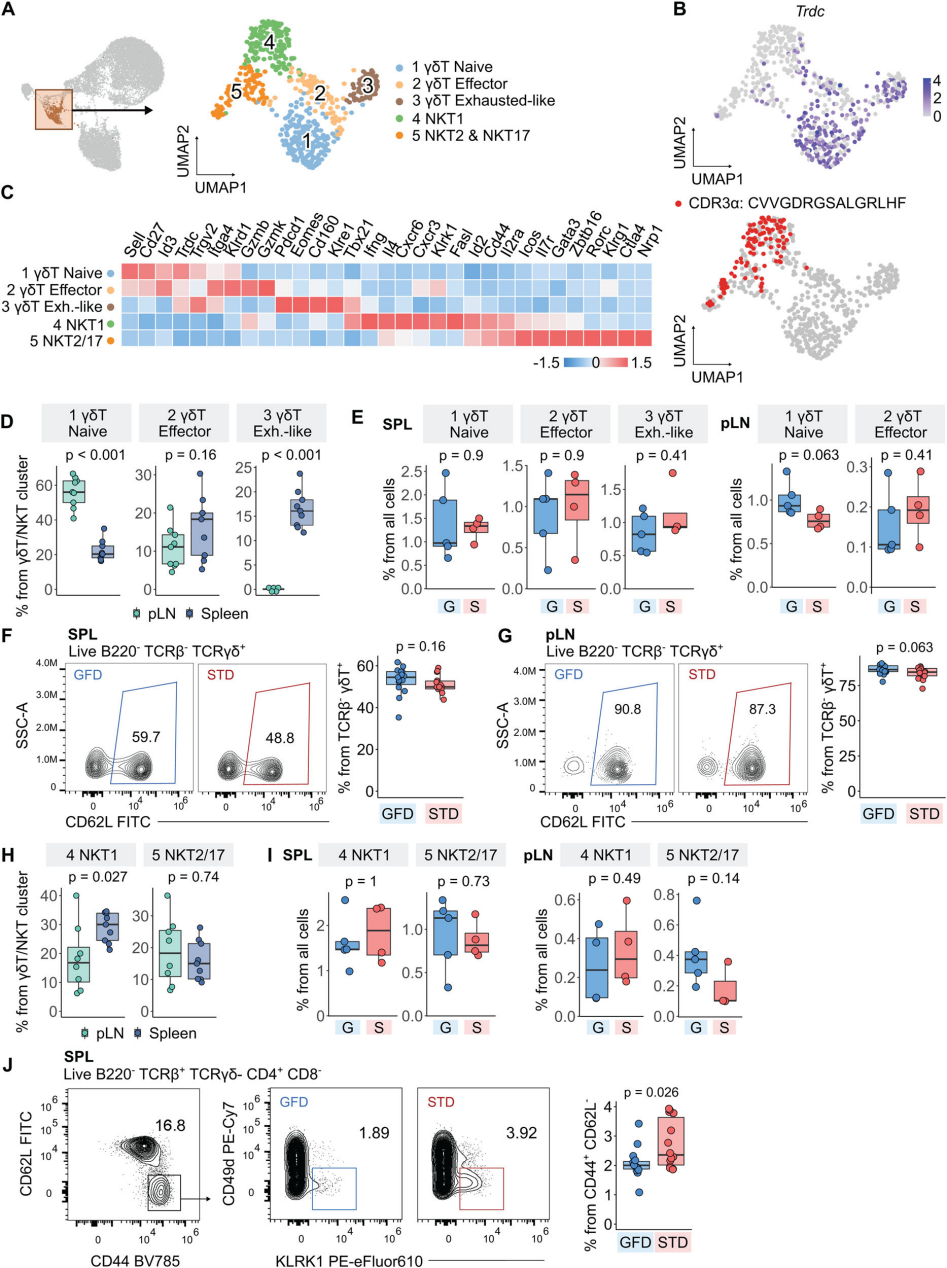
Characterization of γδT cells and NKT cells. (A–E) Reclustering of γδT /NKT cluster from the UMAP projection shown in Figure 1D. The selected cluster was extracted from the dataset and subjected to new normalization, scaling, and dimensional reduction. *n* = 589 cells from nine mice in one scRNAseq experiment. (A) The UMAP projection from the reclustering of γδT /NKT cells showing the clusters used for reclustering (left) and the five new clusters formed (right). Cells are colored by manual annotations. (B) The same UMAP projection as in (A) showing the expression of Trdc gene (upper) and the canonical NKT CDR3α sequence (lower). (C) Heatmap of marker genes that characterize clusters presented in (A). Color represents the column‐scaled *z*‐score of the average expression of a gene in a cluster. *n* = 9 mice from one scRNAseq experiment. (D) Quantification of abundance of clusters representing subpopulations of γδT as shown in (A) in the two analyzed organs. Plotted are percentages of cells in the specified cluster from all cells. *p*‐values were determined by the two‐tailed Mann–Whitney test without correction for multiple comparison. (E) Quantification of abundance of γδT cell clusters from scRNAseq data shown in (A) in mice housed on a gluten‐free diet (G, blue) or standard diet (S, red) in spleen (left) and pLN (right). Plotted are percentages of cells in the specified cluster from all cells. *p*‐values were determined by the two‐tailed Mann‐Whitney test. (F–G) Flow cytometry analysis of manually gated TCRβ^‐^ TCRγδ^+^ CD62L^+^ cells in spleen (F) and pLN (G) Left—representative gating of the population in one mouse housed on gluten‐free diet (GFD, blue) and one mouse housed on standard diet (STD, red). Right—quantification of the percentage of TCRγδ^+^ CD62L^+^ T cells in mice on GFD and on STD. *p*‐values were determined by a two‐tailed Mann–Whitney test. *n* = 29 mice from three independent experiments. (H) Quantification of abundance of clusters representing subpopulations of NKT cells as shown in (A) in the two analyzed organs. Plotted are percentages of cells in the specified cluster from all cells. *p*‐values were determined by a two‐tailed Mann–Whitney test. *n* = 9 mice from one scRNAseq experiment. (I) Quantification of abundance of NKT cell clusters from scRNAseq data shown in (A) in mice housed on a gluten‐free diet (blue) or standard diet (red) in spleen (left) and pLN (right). Plotted are percentages of cells in the specified cluster from all cells. *p*‐values were determined by a two‐tailed Mann–Whitney test. *n* = 9 mice from one scRNAseq experiment. (J) Flow cytometry analysis of manually gated NKT1 cells in spleen Left—representative gating of the population in one mouse housed on a gluten‐free diet (GFD, blue) and one mouse housed on a standard diet (STD, red). Right—quantification of the percentage of NKT1 cells in mice on GFD and on STD. *p*‐values were determined by a two‐tailed Mann–Whitney test. *n* = 29 mice from three independent experiments. In the boxplots, the line represents the median, the hinges correspond to the first and third quartiles, and the whiskers represent 1.5× interquartile range. CDR—complementarity determining region, Exh.‐like—exhausted‐like, G or GFD—gluten‐free diet, NKT—natural killer cells, pLN—pancreatic lymph node, SPL—spleen, S or STD—standard diet, UMAP—uniform manifold approximation and projection.

As NKT and γδT cells were previously proposed as key players in the development of diabetes in NOD mice [33, 34, 35, 36], we inspected the unconventional T cells in greater depth. Since the global scRNAseq analysis revealed γδT cells and NKT cells as a single cluster, we subclustered them and identified three separate subsets of γδT cells and two clusters of NKT cells (Figure 4A–C).

The markers of the γδT cell clusters suggested that γδT cells from cluster 1 had a naïve phenotype (*Sell, Cd27*), γδT cells from cluster 2 expressed effector and cytotoxic molecules (*Itga4*, *Gzmb*), and γδT cells from cluster 3 cells resembled an exhaustion‐like phenotype (*Pdcd1*, *Eomes*, *Cd160*, *Klre1*) [37] (Figure 4C). Whereas naïve γδT cells were enriched in the pLN, the exhausted‐like γδT cells were present exclusively in the spleen (Figure 4D). The naïve γδT cells were slightly more frequent in the pLN of the GFD‐fed mice (Figure 4E), suggesting that GFD might prevent the formation of effector γδT cells, which were previously shown to contribute to the autoimmune diabetes induction [33]. Using FC, we confirmed that naïve CD62L^+^ γδT cells were slightly enriched in the pLNs and spleen of GFD‐fed mice in comparison to standard diet (Figure 4F,G; Figure S5G), although the differences were not significant and relatively subtle.

NKT cells from cluster 4 expressed transcription factor *Tbx21* (Tbet), high levels of *Ifng*, and tissue homing markers (*Cxcr3*, *Cxcr6*), which identified these cells as NKT1 cells [38]. On the other hand, NKT cells from cluster 5 expressed typical markers of NKT2 and NKT17 cells, such as transcription factors *Rorc* (RORγt) and *Gata3*, and genes encoding inhibitory receptors (*Pdcd1, Ctla4*) (Figure 4C). NKT1 cells were more abundant in the spleen than in the pLNs, while NKT2/17 cells had similar frequencies in both organs (Figure 4H). The mice on a GFD diet showed a slightly increased frequency of the NKT2/17 cluster subset and slightly lower frequency of the NKT1 cells (Figure 4I). Based on the gene expression profile of NKT1 cells (Figure S5H), we gated NKT1 cells in FC experiments as TCRβ^+^ TCRγδ^−^ CD4^+^ CD8^−^ CD44^+^ CD62L^−^ CD49d^−^ KLRK1^+^. Consistently with scRNAseq data, we saw a lower abundance of NKT1 cells in animals on GFD in the spleen (Figure 4J; Figure S5I,J).

Overall, these data suggested that the GFD has an impact on unconventional T‐cell subsets as it likely contributes to the maintenance of the naïve state of γδT cells and promotes the NKT2/17 cells at the expense of NKT1 cells.

### 2.5 The Diets Differentially Regulate Signaling by Common Gamma Chain Cytokines and Type I Interferons

**FIGURE 5:**
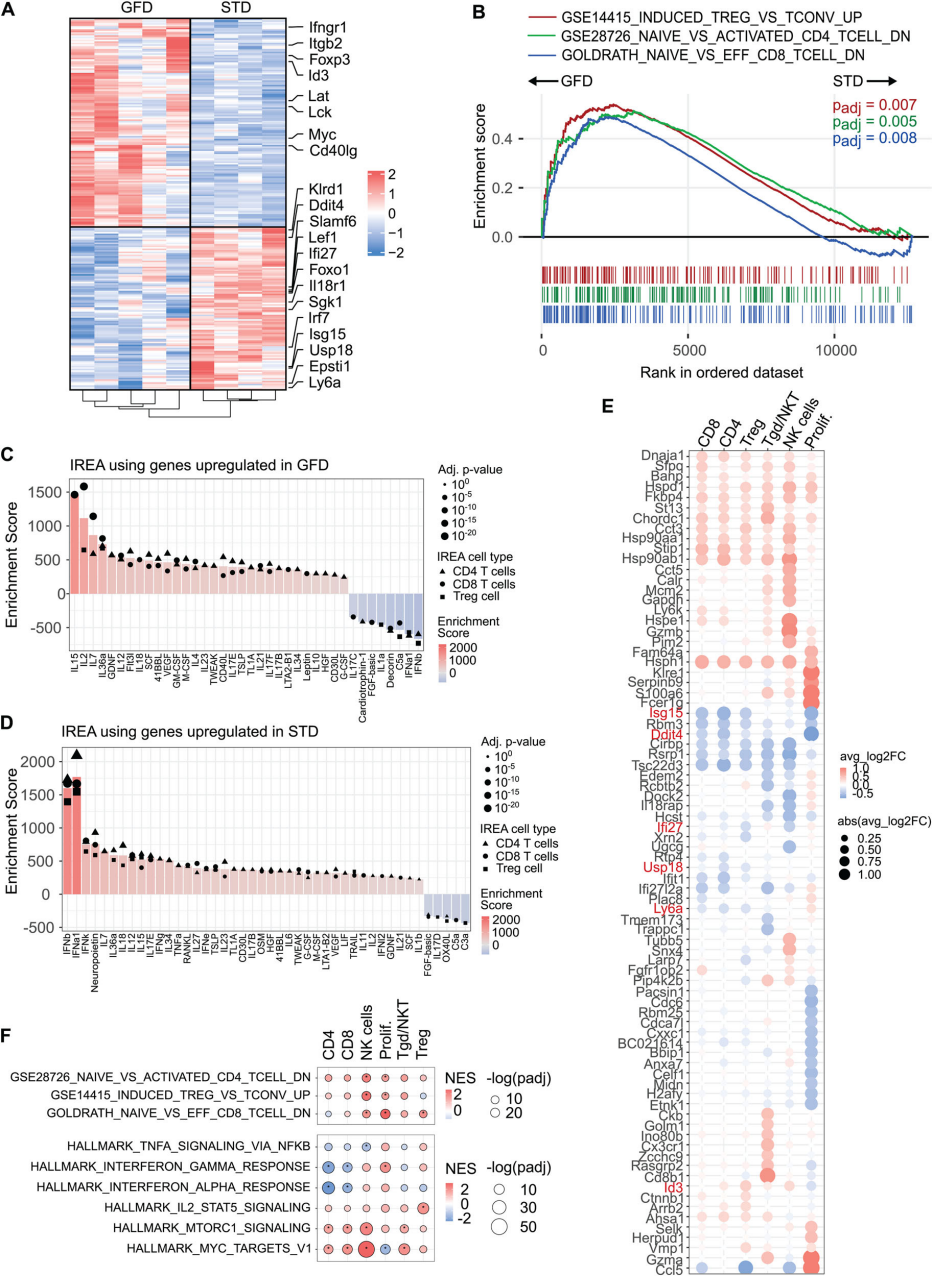
Differential expression analysis between STD and GF diet. Differentially expressed genes were identified between GFD and STD mice from the scRNAseq. (A) Heatmap showing DEG in the whole dataset processed in a pseudobulk manner. Color denotes z‐scores of average expression of the gene in one mouse across tissues and populations. *n* = 9 mice from one scRNAseq experiment. (B) Gene set enrichment analysis (GSEA) using previously published gene sets. Genes that are enriched in GFD mice are positioned on the left, while genes that are enriched in STD mice are positioned on the right. (C, D) Immune response enrichment analysis (IREA) using differentially expressed genes from global comparison of GFD versus STD mice. Genes were compared with published signatures obtained by in vivo activation of different cell types with individual cytokines. (C) IREA using genes upregulated in GFD. (D) IREA using genes upregulated in STD. Bars show the mean of the three IREA cell types tested. (E) Dot plot showing the genes, which were identified as the top 20 DEG upregulated in GFD compared with STD in any of the six populations. The size of the dots represents the percentage of expressing cells, and the color represents the direction of change. (F) GSEA in the six subpopulations comparing the pathway enrichment in GFD vs. STD. The size of the dots represents adjusted *p*‐values, and color represents the normalized enrichment score (NES). The red color indicates enrichment in GFD, and the blue color indicates enrichment in STD. GFD—gluten‐free diet, GSEA—gene set enrichment analysis, IREA—immune response enrichment analysis, NES—normalized enrichment score, NKT—natural killer cells, Prolif.—proliferating cells, STD—standard diet, Treg—Regulatory T cells.

Since our proportionality analysis suggested rather multiple small effects of the GFD, in the next step, we performed an orthogonal analysis of the global gene expression differences between the GFD‐fed and STD‐fed mice. The pseudobulk comparison of the samples from both tissues revealed 247 differentially expressed genes (DEG), among which 139 genes were upregulated in the GFD‐fed mice, and 108 genes were upregulated in mice on the STD (Figure 5A; Table S2). DEGs upregulated in GFD included genes associated with conventional T cells and antigen‐induced T‐cell signaling (*Lck, Lat*), cell activation and proliferation (*Myc, Ifngr1, Cd40lg*), and regulatory T cells (*Foxp3, Id3*). On the contrary, mice with STD showed increased expression of genes associated with naïve and memory T cells (*Lef1, Foxo1, Slamf6, Klf3, Klf7*), activation of NK cells (*Klrd1, Il18r1*), and type 1 interferon (IFN) response (*Irf7, Ifi27, Usp18, Isg15*). In the STD‐fed mice, we observed dysregulation of genes that are known to be regulated by insulin signaling (*Ddit4*, *Sgk1*). To further analyze the gene expression differences, we performed gene set enrichment analysis (GSEA) using published gene sets associated with particular phenotypes (Figure 5B). This analysis revealed a significant upregulation of Treg, effector CD8^+^ T‐cell, and activated CD4^+^ T‐cell signature genes in GFD‐fed mice, which is essentially in line with our single‐cell‐based analysis.

To study a potential diet‐induced dysregulation of the cytokine milieu, we implemented a recently developed Immune Response Enrichment Analysis (IREA), which allows for comparison of DEGs to those observed upon controlled in vivo administration of any of 86 cytokines [39]. We determined that the responses observed in GFD mice seem to be determined mostly by common γ chain cytokines IL‐15, IL‐2, and IL‐7 (Figure 5C), which control T‐cell homeostasis, expansion, and activation. In contrast, the gene signatures observed in STD‐fed mice are highly similar to those occurring upon type I IFN administration (Figure 5D).

Overall, the global gene expression signature of T cells in the GFD‐fed mice is biased toward markers of effector and Treg and is enriched for genes responding to IL‐2, IL‐7, and IL‐15. The global signature in the STD‐fed mice is biased toward naïve T‐cells and IFN signaling.

Finally, we studied whether the observed global changes are shared across different cell populations or predominantly driven by a specific subset. Analysis of DEG between animals on GFD and STD revealed that most genes were consistently up‐ or downregulated across different subsets (Figure 5E), including main markers shown in Figure 5A (*Isg15, Ddit4, Ifi27, Usp18, Ly6a, Id3*). On the other hand, subset‐specific dysregulation was observed in genes with expression restricted to a particular subset, such as *Gzmb* in NK cells or *Cdc6* in proliferating cells (Figure 5E).

GSEA in different populations showed that the shift toward an effector phenotype in GFD is primarily driven by NK cells and proliferating cells (Figure 5F). In addition, GFD was characterized by the downregulation of genes responsive to IFNγ and IFNα in conventional CD4^+^ and CD8^+^ T cells, the upregulation of STAT5 signaling in Tregs, and the upregulation of pathways associated with proliferation in CD4^+^ T cells, CD8^+^ T cells, NK cells, and unconventional subsets (Figure 5F). These orthogonal analyses are consistent with the global IREA analysis and further support the observation of an enhanced effector phenotype in GFD.

## 3 Discussion

The preventive effect of GFD on the development of autoimmune diabetes in NOD mice has been shown consistently for the last twenty years [15, 16, 17, 20], with one exceptional study that did not observe this effect [40]. However, our understanding of the mechanism is still limited and hindered by controversial data in some areas, such as whether gluten itself is the disease‐permissive component in autoimmunity [17, 27, 41], whether the effect might be related to other components within the wheat diet, for example, wheat amylase trypsin inhibitors [42], or whether the disease is mediated indirectly by intestinal microbiota, which adapts to dietary changes [12, 20, 26]. In this study, we focused on the changes that gluten induces in the primary causative cells, conventional and unconventional T cells, which initiate the disease by autoimmune attack of self‐tissue.

Our transcriptomic profiling at the single‐cell level showed that the STD did not induce more activated T cells than GFD. The STD was even associated with a more naïve phenotype. This seems to be contradictory to the previous studies suggesting that dietary gluten caused proinflammatory Th1‐ or Th17‐polarizing conditions and activation of T cells and NK cells in lymphoid organs [43, 44, 45]. However, these studies mostly focused on the intestinal mucosal immune compartment [43, 45]. The evidence for the inflammatory effects of gluten at the systemic level is much weaker. For instance, serum IFNγ levels were not different between NOD mice on STD and GFD diets [45]. Overall, we conclude that the GFD does not induce robust systemic activation of the lymphoid compartment.

Our observation that T1D might be associated with a rather naïve state of the immune system, that is, the SFD conditions in our case, is in line with the hygienic hypothesis, which explains the development of certain atopic and autoimmune diseases, including T1D, by insufficient priming of the immune responses in early childhood [46, 47]. Recently, our analysis of T cells from the peripheral blood of children with T1D revealed a deficit in effector populations in multiple cohorts [48], showing that this finding is relevant for human disease. We also observed that the STD induced Type I IFN signatures, which is in line with the observed pathological role of Type I IFN in the initiation stage of NOD diabetes [49].

In addition to the global changes, we focused on Tregs and unconventional T cells—two subsets that were previously connected with diabetes pathology in NOD mice [33, 34, 50, 51, 52, 53]. Whereas one previous study proposed that the GFD leads to a higher frequency of Tregs among CD4^+^ T cells in pLNs [17], another study did not observe this effect [44]. Accordingly, we observed a very slight but nonsignificant increase in Tregs in the spleen and pLN. However, our detailed analysis of Treg subpopulations indicated that a specific Treg subset characterized by cytotoxic markers was enriched in the GFD‐fed mice. Cytotoxic Tregs express ICOS and might thus be similar to previously described ICOS^hi^ Tregs [54, 55, 56], which are found in the pancreas of NOD mice and are specific to the antigens from the pancreatic islets. In a previous study, ICOS^hi^ Tregs effectively blocked diabetes in adoptive transfer experiments in an ICOS‐dependent manner [54]. A similar phenotype of cytotoxic Tregs in our datasets suggests that these splenic Tregs might have the same protective ability and might be precursors of pancreatic ICOS^hi^ Treg. In addition, our GSEA analysis revealed enhanced STAT5 signaling in Treg of GFD mice, reinforcing previous findings that highlight the critical role of IL‐2R signaling in diabetes prevention in NOD mice [57, 58].

The role of unconventional T‐cell subsets in the development of diabetes in NOD mice is unclear. γδT cells are more abundant in NOD mice than in other nondiabetic strains [59, 60], and in the context of T1D, they are considered regulatory as intranasal administration of insulin led to the induction of disease‐protective CD8^+^ γδT cells [61, 62]. With respect to GFD, γδT cells were increased in BALB/C mice fed the GFD [44], and diabetes‐preventive intranasal administration of gliadin, a major gluten component, to NOD mice led to an increased proportion of γδT cells, preferentially in mucosal lymphoid organs [63]. The tolerogenic effects of γδT cells are mediated by TGF‐β production [34]. On the other hand, CD27^‐^ effector γδT cells were reported to contribute to islet destruction by the production of IL‐17 [33]. We observed that CD27^+^ naïve‐like γδT cells were enriched in the GFD mice, which might contribute to their reduced susceptibility to T1D.

NKT cells in the GFD‐fed mice were biased toward the type 2/17 phenotype, whereas the STD‐fed mice were enriched for NKT1 cells. While it is established that NKT cells can suppress the development of T1D in NOD mice [51, 64, 65], it is not clear which particular subtypes mediate this protection, and further research in this field is warranted. We showed that NKT1 cells express KLRK1, which encodes the activating NK‐cell receptor NKG2D. Blockade of NKG2D prevented diabetes development and progression in previous studies [66, 67], suggesting that it might suppress the function of pathogenic NKT1 cells and promote regulatory type 2/17 NKT cells.

In conclusion, our comprehensive profiling of T‐cell populations and gene expression in NOD mice reveals that the GFD induces a significant immune modulation characterized by increased frequencies of effector and activated regulatory T cells and modulates the differentiation of γδT cells and NKT cells. Although the effect of the GFD on the individual T‐cell subsets was rather subtle, collectively, these changes might lead to a substantially decreased risk of spontaneous T1D development. These findings enhance our understanding of the environmental impact on autoimmune diabetes and potentially open new avenues for therapeutic intervention in T1D.

## 4 Data Limitations and Perspectives

The studies of environmental factors in NOD mice are hindered by incomplete penetrance of the disease, which is further complicated by differences in particular breedings, animal facilities, and dietary compositions, leading to inconsistent results. Accordingly, the effect of the two diets in NOD mice is nonbinary in relation to the T1D incidence. Some STD‐fed mice are still protected, and some GFD‐fed mice still develop the disease. Since our analyses were performed before the eventual T1D onset, we do not know which particular animals would have developed T1D. This phenomenon decreases the statistical power of our results, so further studies are needed to recapitulate our findings in larger cohorts and distinct time points in the course of the disease development.

With our detailed single‐cell transcriptomic approach, we did not reveal a single substantial difference in the lymphocyte compartment between mice fed with STD and GFD. Instead, we observed multiple small differences, which collectively contribute to the beneficial effect of GFD on the diabetes incidence. However, we did not determine whether each of these individual GFD‐driven changes actually contributes to the protective effect of GFD or is merely an association.

It is currently unclear whether the GFD‐induced changes described here are specific to NOD mice or general to all murine strains or even to other mammalian species. Although our interpretation of the data presented here would not be changed, this eventual knowledge could contribute to the understanding of why NOD mice, but not other laboratory‐inbred strains, are susceptible to diabetes.

## 5 Materials and Methods

### 5.1 Animals, Diets, and Diabetes Incidence

NOD/ShiLtJ mice were purchased from The Jackson Laboratory (Bar Harbor, ME), NOD‐SCID mice were obtained from Taconic (Albany, NY), kept in specific‐pathogen‐free (SPF) conditions according to the FELASA guidelines at the animal facilities of the Institute of Microbiology, CAS, under standard light‐ and climate‐controlled conditions and had ad libitum access to diets and water. Experiments were carried out in line with the EU legislation on the use of experimental animals—EU Directive 2010/63, and were approved by the Committee for the Protection and Use of Experimental Animals of the Institute of Microbiology, CAS; approval ID: 24‐2022‐P.

The breeding pairs of NOD mice were fed the standard 5c (STD) or gluten‐free 5d (GFD) 1434 diets (Altromin, Lage, Germany), thus, all the experimental female NOD mice used for the observation of the development of spontaneous diabetes incidence were exposed to the GFD (or STD) diets prenatally. The composition of the diets has been described previously [27]. In brief, these open‐formula Altromin diets were designed so that there is the same content of milk and soybean proteins, whereas the gluten‐containing ingredients were replaced with meat protein while keeping as similar as possible (with respect to the open‐formula character of the diets) the content of total protein, fat, fiber and minerals. The total protein content of the GFD and STD diets is 22.7% and 22.9%, respectively. The two diets were equally supplemented with vitamins.

Groups of 16 NOD females were used for the monitoring of the natural course of the development of diabetes on the GFD and STD. Screening NOD mice for diabetes incidence was carried out from the age of 10 weeks until the age of 310 days. The diabetes onset was monitored weekly from tail vein blood with the glucometer Freestyle Lite (Abbott Diabetes Care Ltd., Witney, UK), and the diagnosis of diabetes was based on two consecutive blood glucose readings >12 mM in 3 days. The first reading was used as the date of diabetes onset. None of the experimental animals died from other causes.

For adoptive transfer experiments, 8‐week‐old NOD‐SCID female mice, fed a standard Altromin diet, were used as the recipients of 5 × 10^6^ live diabetogenic splenocytes isolated and pooled from 10 prediabetic 12‐week‐old NOD females born on the GFD or STD Altromin diets. Erythrocytes were lysed with red blood cell lysing buffer (Sigma), and cells were washed twice in PBS and injected i.p. in a final volume of 200 µL of PBS. The diabetes onset was monitored weekly for a period of 12 weeks from tail vein blood with the glucometer Freestyle Lite (Abbott Diabetes Care Ltd), and animals were considered diabetic when blood glucose readings >12 mM.

### 5.2 Single‐Cell RNA Sequencing

Six to eight‐week‐old female mice were sacrificed by cervical dislocation. Single‐cell suspensions from spleens were lysed with 1x ACK buffer (1.5 M NH_4_Cl, 100 mM KHCO_3_, 10 mM Titriplex III) for 3 min on ice to remove erythrocyte contamination. Single‐cell suspensions from pLN and lysed spleens were labeled with the LIVE/DEAD Fixable Near‐IR Dead Cell Stain Kit (Invitrogen #L34975), anti‐CD45R/B220 (clone RA3‐6B2, Biolegend #103248) and purified Fc receptors‐blocking anti‐CD16/32 (clone 2.4G2, BD Pharmingen # 553141) and one of the following hashtag antibodies: Mouse hashtag #1 (ACCCACCAGTAAGAC, Biolegend #155861), Mouse hashtag #2 (GGTCGAGAGCATTCA, Biolegend #155863), Mouse hashtag #3 (CTTGCCGCATGTCAT, Biolegend #155865), Mouse hashtag #4 (AAAGCATTCTTCACG, Biolegend #155867), Mouse hashtag #5 (CTTTGTCTTTGTGAG, Biolegend #155869), Mouse hashtag #6 (TATGCTGCCACGGTA, Biolegend #155871), Mouse hashtag #7 (GAGTCTGCCAGTATC, Biolegend #155873), Mouse hashtag #8 (TATAGAACGCCAGGC, Biolegend #155875), Mouse hashtag #9 (TGCCTATGAAACAAG, Biolegend #155877) for 30 minutes on ice. Hashtags #1‐5 were used for mice on GFD, while hashtags #6‐9 were for mice fed with STD. A total of 10,000 hashtag‐labeled CD45R/B220‐negative live lymphocytes were sorted using FACSAria III (BD Bioscience). Cells from each tissue were mixed together in one tube, washed with PBS/0.05% BSA, and counted using the TC20 Automated Cell Counter (#1450102, Bio‐Rad). The viability of the cells preloading was higher than 90%.

Cells were loaded onto a 10× Chromium machine (10× Genomics) aiming at the yield of 1500 cells per sample. cDNA libraries were prepared using the Feature Barcode technology for Cell Surface Protein protocol (#CG000186 Rev D) with the Chromium Single Cell 5’ Library & Gel Bead and Chromium Single Cell 5' Feature Barcode Library kits (10× Genomics, #PN‐1000014, #PN‐1000020, #PN‐1000080, #PN‐1000009, #PN‐1000084, #PN‐1000071) according to the manufacturer's instructions. Sequencing was performed on the NovaSeq 6000 platform (Illumina).

### 5.3 Analysis of scRNAseq Data

Raw sequencing data were mapped using Cell Ranger (10× Genomics, v5.0.1) [68] with chemistry and length‐r1 parameters being set to SC5P‐R2 and 35, respectively (other parameters by default), and the mouse reference genome “Mus_musculus_nodshiltj.NOD_ShiLtJ_v1.102” [69] for gene expression libraries (hashtag sequences were aligned to hashtag reference during the same step), and the default parameters and International ImMunoGeneTics Information System reference [70] for the immune receptor repertoire profiling.

Initially, cells with less than 200 transcripts and/or more than 15% of transcripts mapping to mitochondrial genes and cells identified as doublets by V(D)J sequences (having more than 2 TCRα or 1 productive TCRβ or 2 total TCRβ sequences) were removed. Hashtag counts were used to identify the source sample of each cell, with hashtag count thresholds being determined from histograms of a number of cells’ count per hashtag count. Cells that were marked by multiple hashtags or none at all were removed. Mitochondrial genes, ribosomal genes, genes encoding TCR chains, and genes detected in less than three cells in total were excluded. For the downstream analysis, datasets were annotated with the ImmGen Immune Dataset using the SingleR package (v1.0.1) [71] and filtered to contain only non‐B‐cell lymphocyte subpopulations (T cells, NK cells, NKT, γδT, ILC) with high‐quality measures (more than 750 detected genes and less than 7.5% of mitochondrial genes expressed). Merging of the two wells, log normalization (scale factor = 1 × 10^4^), scaling, identification of variable features (1000 variable features), dimensional reduction (PCA with 10 principal components and UMAP), identification of nearest neighbors and Louvain clustering were performed using the Seurat R package v4.3.0 [72] on R v4.1.2. Differentially expressed genes were identified using the Mann–Whitney test with the default Seurat criteria and filtering for markers with positive log2 fold change values. Heatmaps were constructed using the R package pheatmap (v1.0.12).

GSEA pathways were processed using gene sets from datasets from GSE14415 [73], GSE28726 [74], and Msigdb #M3035 [75] with the R package fgsea (v1.20.0). Fold changes from STD versus GFD mice were calculated with the Seurat function FoldChange. IREA analysis was processed using the online IREA tool [39]. As input, we used the DEG from the STD versus GFD comparison. Output data were downloaded as csv files and processed using ggplot2 (v3.3.5).

The code for the cell filtration to hashtags, barcode extraction, and whole downstream analysis, including V(D)J analysis and reanalysis of public data, is accessible on GitHub (https://github.com/Lab‐of‐Adaptive‐Immunity/NOD_scRNAseq).

### 5.4 Antibodies and Reagents

#### 5.4.1 Extracellular Markers

Anti‐CD4 BV650 (clone RM4‐5, Biolegend #100545) or AF700 (clone RM4‐5, Biolegend #100536), anti‐CD8a FITC (clone 53–6.7, eBioscience #11‐0081‐81) or BV421 (clone 53–6.7, Biolegend # 100753) or BV510 (clone 53–6.7, Biolegend # 100752), anti‐CD25 BV605 (clone PC61, Biolegend #102036), anti‐CD44 BV785 (clone IM7, Biolegend #103059) or BV650 (clone IM7, Biolegend #103049), anti‐CD45R/B220 BV510 (clone RA3‐6B2, Biolegend #103248) or AF700 (clone RA3‐6B2, Biolegend #103232), anti‐CD49d PE‐Cy7 (clone R1‐2, Biolegend #103618), anti‐CD62L FITC (clone MEL‐14, Biolegend #104406) or AF700 (clone MEL‐14, Biolegend #104426), anti‐CD69 PerCP‐Cy5.5 (clone H1.2F3, BD Pharmingen #551113), anti‐CD103 BV421 (clone 2 E7, Biolegend #121422), anti‐CD137 PE (clone 17B5, Biolegend #106106), anti‐CD314/KLRK1 PE‐eFluor610 (clone CX5, eBioscience #61‐5882‐82), anti‐CX3CR1 BV605 (clone SA011F11, Biolegend #149027), anti‐KLRG1 BV785 (clone 2F1/, Biolegend #138429), anti‐TCR β Chain APC (clone H57‐597, Biolegend #109212), anti‐TCR γδ BV421 (clone GL3, Biolegend #118120).

#### 5.4.2 Viability

LIVE/DEAD fixable near‐IR dead cell stain kit (Invitrogen #L34975).

#### 5.4.3 Intracellular Markers

Anti‐FOXP3 PE‐Cy7 (clone 150D, Biolegend #320012) or AF488 (clone FJK‐16s, eBioscience #25‐5773‐82), anti‐T‐bet PerCP‐Cy5.5 (clone 4B10, Biolegend #644806), anti‐Ki‐67 AF647 (clone 11F6, Biolegend #151206), anti‐Helios AF647 (clone 22F6, BD Pharmingen #563951), anti‐TCF‐7/TCF‐1 PE (clone S33‐966, BD Pharmingen #564217), anti‐EOMES AF488 (clone Dan11mag, eBioscience #53‐4875‐80).

### 5.5 Flow Cytometry

For the surface staining, cell preparation was the same as described above for the scRNAseq. Cells were incubated with diluted antibodies for 30 min on ice immediately after isolation. For staining of intracellular markers and transcription factors, cells were stained for 20 min on ice with LIVE/DEAD fixable near‐IR dead cell stain kit (Invitrogen #L34975) immediately after isolation, and then fixed and permeabilized using the eBioscience Foxp3/transcription factor staining buffer set (Invitrogen #00‐5523‐00) according to the manufacturer's instructions, washed, and stained overnight with antibodies for intracellular markers in 4°C. On the next day, cells were washed and incubated for 1 h on ice with extracellular markers. To prevent unspecific binding to Fc receptors, purified anti‐CD16/32 (clone 2.4G2, BD Pharmingen # 553141) was added to all staining mixes.

Flow cytometry was carried out with Cytek Aurora flow cytometer, configuration 4L 16V‐14B‐10YG‐8R (Cytek). Data were analyzed using FlowJo software v10.10 (BD Life Sciences).

### 5.6 Statistical Analysis

The statistical analysis was performed using tests indicated in the Figure legends using R v4.1.2 or GraphPad Prism 5.0. Quantitative scRNAseq and FC data were tested using the nonparametric Mann–Whitney test without a correction for multiple comparisons or, for a comparison of more than two sample groups, the Kruskal–Wallis test with Dunn's posttest if required. The T1D incidence data were analyzed using the log‐rank (Mantel–Cox) Test. Typically, two‐tailed tests were performed. One‐tailed tests were used in exceptional cases when the results from scRNAseq were directly verified by FC. The number of biological replicates (mice) is indicated in the respective Figure legends.

## Author Contributions

Veronika Niederlova, Radka Cisarova, and Barbora Drabonova performed experiments. Veronika Niederlova and Juraj Michalik analyzed the data. Veronika Niederlova and Ondrej Stepanek wrote the manuscript. Ondrej Stepanek and David Funda designed and supervised the study and obtained funding. All authors approved the final manuscript.

## Ethics Statement

All animal experiments were carried out in line with the EU legislation on the use of experimental animals—EU Directive 2010/63 and were approved by the Committee for the protection and use of experimental animals of the Institute of Microbiology, Czech Academy of Sciences; approval ID: 24‐2022‐P.

## Conflicts of Interest

The authors declare no conflicts of interest.

### 1 Peer Review

The peer review history for this article is available at https://publons.com/publon/10.1002/eji.202451559.

## Supporting information



Supporting information

Supporting information

Supporting information

## Data Availability

All scRNAseq data analyzed in this study, as well as the scripts used for the analysis, are available without restrictions on GitHub (https://github.com/Lab‐of‐Adaptive‐Immunity/NOD_scRNAseq). The scRNAseq data generated in this study were deposited in the Sequence Read Archive SRA under the accession number PRJNA1157582.

## References

[1] G. A. Gregory , T. I. G. Robinson , S. E. Linklater , et al., “Global Incidence, Prevalence, and Mortality of Type 1 Diabetes in 2021 With Projection to 2040: A Modelling Study,” The Lancet Diabetes & Endocrinology 10, no. 10 (2022): 741–760. 36113507 10.1016/S2213-8587(22)00218-2

[2] M. S. Anderson and J. A. Bluestone , “THE NOD MOUSE: A Model of Immune Dysregulation,” Annual Review of Immunology 23 (2005): 447–485. 10.1146/annurev.immunol.23.021704.115643 15771578

[3] J. Chiou , R. J. Geusz , M.‐L. Okino , et al., “Interpreting Type 1 Diabetes Risk With Genetics and Single‐cell Epigenomics,” Nature 594, no. 7863 (2021): 398–402. 34012112 10.1038/s41586-021-03552-w PMC10560508

[4] V. Forgetta , D. Manousaki , R. Istomine , et al., “Rare Genetic Variants of Large Effect Influence Risk of Type 1 Diabetes,” Diabetes 69, no. 4 (2020): 784–795. 32005708 10.2337/db19-0831 PMC7085253

[5] C. C. Robertson , J. R. J. Inshaw , S. Onengut‐Gumuscu , et al., “Fine‐mapping, Trans‐ancestral and Genomic Analyses Identify Causal Variants, Cells, Genes and Drug Targets for Type 1 Diabetes,” Nature Genetics 53, no. 7 (2021): 962–971. 34127860 10.1038/s41588-021-00880-5 PMC8273124

[6] S. V. Gearty , F. Dündar , P. Zumbo , et al., “An Autoimmune Stem‐Like CD8 T Cell Population Drives Type 1 Diabetes,” Nature 602, no. 7895 (2022): 156–161. 34847567 10.1038/s41586-021-04248-x PMC9315050

[7] K. C. Herold , T. Delong , A. L. Perdigoto , N. Biru , T. M. Brusko , and L. S. K. Walker , “The Immunology of Type 1 Diabetes,” Nature Reviews Immunology 24, no. 6 (2024): 435–451. 10.1038/s41577-023-00985-4 PMC7616056 38308004

[8] M. Bettini and M. L. Bettini , “Function, Failure, and the Future Potential of Tregs in Type 1 Diabetes,” Diabetes 70, no. 6 (2021): 1211–1219. 34016597 10.2337/dbi18-0058 PMC8275894

[9] V. Hyttinen , J. Kaprio , L. Kinnunen , M. Koskenvuo , and J. Tuomilehto , “Genetic Liability of Type 1 Diabetes and the Onset Age Among 22,650 Young Finnish Twin Pairs: A Nationwide Follow‐up Study,” Diabetes 52, no. 4 (2003): 1052–1055. 12663480 10.2337/diabetes.52.4.1052

[10] M. J. Redondo , L. Yu , M. Hawa , et al., “Heterogeneity of Type I Diabetes: Analysis of Monozygotic Twins in Great Britain and the United States,” Diabetologia 44, no. 3 (2001): 354–362. 11317668 10.1007/s001250051626

[11] B. Gong , W. Yang , Y. Xing , Y. Lai , and Z. Shan , “Global, Regional, and National Burden of Type 1 Diabetes in Adolescents and Young Adults,” Pediatric Research (2024). 10.1038/s41390-024-03107-5 PMC12015108 38443523

[12] V. Neuman , O. Cinek , D. P. Funda , et al., “Human Gut Microbiota Transferred to Germ‐free NOD Mice Modulate the Progression towards Type 1 Diabetes Regardless of the Pace of Beta Cell Function Loss in the Donor,” Diabetologia 62, no. 7 (2019): 1291–1296. 31025045 10.1007/s00125-019-4869-2

[13] L. Wen , R. E. Ley , P. Y. Volchkov , et al., “Innate Immunity and Intestinal Microbiota in the Development of Type 1 Diabetes,” Nature 455, no. 7216 (2008): 1109–1113. 18806780 10.1038/nature07336 PMC2574766

[14] S. Wilberz , H. J. Partke , F. Dagnaes‐Hansen , and L. Herberg , “Persistent MHV (mouse hepatitis virus) Infection Reduces the Incidence of Diabetes Mellitus in Non‐Obese Diabetic Mice,” Diabetologia 34, no. 1 (1991): 2–5. 1647335 10.1007/BF00404016

[15] A. K. Hansen , F. Ling , A. Kaas , D. P. Funda , H. Farlov , and K. Buschard , “Diabetes Preventive Gluten‐free Diet Decreases the Number of Caecal Bacteria in Non‐Obese Diabetic Mice,” Diabetes Metabolism Research and Reviews 22, no. 3 (2006): 220–225. 16355418 10.1002/dmrr.609

[16] D. P. Funda , A. Kaas , T. Bock , H. Tlaskalová‐Hogenová , and K. Buschard , “Gluten‐free Diet Prevents Diabetes in NOD Mice,” Diabetes Metabolism Research and Reviews 15, no. 5 (1999): 323–327. 10585617 10.1002/(sici)1520-7560(199909/10)15:5<323::aid-dmrr53>3.0.co;2-p

[17] C. H. F. Hansen , C. S. Larsen , L. F. Zachariassen , et al., “Gluten‐free Diet Reduces Autoimmune Diabetes Mellitus in Mice Across Multiple Generations in a Microbiota‐independent Manner,” Journal of Autoimmunity 127 (2022): 102795. 35101708 10.1016/j.jaut.2022.102795

[18] S. Schmid , K. Koczwara , S. Schwinghammer , V. Lampasona , A.‐G. Ziegler , and E. Bonifacio , “Delayed Exposure to Wheat and Barley Proteins Reduces Diabetes Incidence in Non‐Obese Diabetic Mice,” Clinical Immunology 111, no. 1 (2004): 108–118. 15093559 10.1016/j.clim.2003.09.012

[19] T. Suzuki , et al., Diabetogenic Effects of Lymphocyte Transfusion on the NOD or NOD Nude Mouse, in Immune‐Deficient Animals in Biomedical Research: 5th International Workshop, Copenhagen, October 1985, J. Rygaard , et al., Editors. 1987, S. Karger (1987). AG. p. 0.

[20] C. H. F. Hansen , Ł. Krych , K. Buschard , et al., “A Maternal Gluten‐free Diet Reduces Inflammation and Diabetes Incidence in the Offspring of NOD Mice,” Diabetes 63, no. 8 (2014): 2821–2832. 24696449 10.2337/db13-1612

[21] V. Neuman , S. Pruhova , M. Kulich , et al., “Gluten‐free Diet in Children With Recent‐onset Type 1 Diabetes: A 12‐month Intervention Trial,” Diabetes, Obesity & Metabolism 22, no. 5 (2020): 866–872. 10.1111/dom.13974 31984648

[22] P. N. Zakharov , H. Hu , X. Wan , and E. R. Unanue , “Single‐cell RNA Sequencing of Murine Islets Shows High Cellular Complexity at all Stages of Autoimmune Diabetes,” Journal of Experimental Medicine 217, no. 6 (2020). 10.1084/jem.20192362 PMC7971127 32251514

[23] K. Hrovatin , A. Bastidas‐Ponce , M. Bakhti , et al., “Delineating Mouse Beta‐cell Identity During Lifetime and in Diabetes With a Single Cell Atlas,” Nature Metabolism 5, no. 9 (2023): 1615–1637. 10.1038/s42255-023-00876-x PMC10513934 37697055

[24] H. Hu , P. N. Zakharov , O. J. Peterson , and E. R. Unanue , “Cytocidal Macrophages in Symbiosis With CD4 and CD8 T Cells Cause Acute Diabetes Following Checkpoint Blockade of PD‐1 in NOD Mice,” PNAS 117, no. 49 (2020): 31319–31330. 33229539 10.1073/pnas.2019743117 PMC7733808

[25] J. L. Collier , K. E. Pauken , C. A. A. Lee , et al., “Single‐cell Profiling Reveals Unique Features of Diabetogenic T Cells in anti‐PD‐1‐induced Type 1 Diabetes Mice,” Journal of Experimental Medicine 220, no. 10 (2023). 10.1084/jem.20221920 PMC10336233 37432393

[26] M. C. Funsten , L. A. Yurkovetskiy , A. Kuznetsov , et al., “Microbiota‐dependent Proteolysis of Gluten Subverts Diet‐mediated Protection Against Type 1 Diabetes,” Cell Host & Microbe 31, no. 2 (2023): 213–227.e9. 36603588 10.1016/j.chom.2022.12.009 PMC9911364

[27] D. P. Funda , A. Kaas , H. Tlaskalová‐Hogenová , and K. Buschard , “Gluten‐free but Also Gluten‐enriched (gluten+) Diet Prevent Diabetes in NOD Mice; the Gluten Enigma in Type 1 Diabetes,” Diabetes Metabolism Research and Reviews 24, no. 1 (2008): 59–63. 17607660 10.1002/dmrr.748

[28] A. Moudra , V. Niederlova , J. Novotny , et al., “Phenotypic and Clonal Stability of Antigen‐Inexperienced Memory‐Like T Cells Across the Genetic Background, Hygienic Status, and Aging,” Journal of Immunology 206, no. 9 (2021): 2109–2121. 10.4049/jimmunol.2001028 PMC7610663 33858960

[29] D. Paprckova , V. Niederlova , A. Moudra , et al., “Self‐reactivity of CD8 T‐cell Clones Determines Their Differentiation Status Rather Than Their Responsiveness in Infections,” Frontiers in immunology 13 (2022): 1009198. 36275704 10.3389/fimmu.2022.1009198 PMC9582129

[30] X. Wang , X. Shen , S. Chen , et al., “Reinvestigation of Classic T Cell Subsets and Identification of Novel Cell Subpopulations by Single‐Cell RNA Sequencing,” Journal of Immunology 208, no. 2 (2022): 396–406. 10.4049/jimmunol.2100581 34911770

[31] R. Bedel , R. Berry , T. Mallevaey , et al., “Effective Functional Maturation of Invariant Natural Killer T Cells Is Constrained by Negative Selection and T‐cell Antigen Receptor Affinity,” PNAS 111, no. 1 (2014): E119–E128. 24344267 10.1073/pnas.1320777110 PMC3890789

[32] M. Ejsing‐Duun , J. Josephsen , B. Aasted , K. Buschard , and A. K. Hansen , “Dietary Gluten Reduces the Number of Intestinal Regulatory T Cells in Mice,” Scandinavian Journal of Immunology 67, no. 6 (2008): 553–559. 18476878 10.1111/j.1365-3083.2008.02104.x

[33] J. G. M. Markle , S. Mortin‐Toth , A. S. L. Wong , L. Geng , A. Hayday , and J. S. Danska , “gammadelta T Cells Are Essential Effectors of Type 1 Diabetes in the nonobese Diabetic Mouse Model,” Journal of Immunology 190, no. 11 (2013): 5392–5401. 10.4049/jimmunol.1203502 PMC3836168 23626013

[34] G. Han , R. Wang , G. Chen , et al., “Interleukin‐17‐producing Gammadelta+ T Cells Protect NOD Mice From Type 1 Diabetes Through a Mechanism Involving Transforming Growth Factor‐beta,” Immunology 129, no. 2 (2010): 197–206. 19824917 10.1111/j.1365-2567.2009.03166.x PMC2814462

[35] S. Sharif , G. A. Arreaza , P. Zucker , et al., “Activation of Natural Killer T Cells by Alpha‐galactosylceramide Treatment Prevents the Onset and Recurrence of Autoimmune Type 1 Diabetes,” Nature Medicine 7, no. 9 (2001): 1057–1062. 10.1038/nm0901-1057 11533711

[36] T. Griseri , L. Beaudoin , J. Novak , et al., “Invariant NKT Cells Exacerbate Type 1 Diabetes Induced by CD8 T Cells,” Journal of Immunology 175, no. 4 (2005): 2091–2101. 10.4049/jimmunol.175.4.2091 16081775

[37] E. Jenkins , T. Whitehead , M. Fellermeyer , S. J. Davis , and S. Sharma , “The Current state and Future of T‐cell Exhaustion Research,” Oxford Open Immunology 4, no. 1 (2023): iqad006. 37554723 10.1093/oxfimm/iqad006 PMC10352049

[38] M. G. Constantinides and A. Bendelac , “Transcriptional Regulation of the NKT Cell Lineage,” Current Opinion in Immunology 25, no. 2 (2013): 161–167. 23402834 10.1016/j.coi.2013.01.003 PMC3647452

[39] A. Cui , T. Huang , S. Li , et al., “Dictionary of Immune Responses to Cytokines at Single‐cell Resolution,” Nature 625, no. 7994 (2024): 377–384. 38057668 10.1038/s41586-023-06816-9 PMC10781646

[40] M. Ø. Mønsted , L. J. Holm , K. Buschard , and M. Haupt‐Jorgensen , “Failure to Replicate the Diabetes Alleviating Effect of a Maternal Gluten‐free Diet in Non‐Obese Diabetic Mice,” PLoS ONE 18, no. 9 (2023): e0289258. 37682921 10.1371/journal.pone.0289258 PMC10490983

[41] V. Neuman , S. Pruhova , M. Kulich , et al., “Changes in the Gut Bacteriome Upon Gluten‐free Diet Intervention Do Not Mediate Beta Cell Preservation,” Diabetologia 66, no. 1 (2023): 241–246. 36194251 10.1007/s00125-022-05805-3

[42] Y. Junker , S. Zeissig , S.‐J. Kim , et al., “Wheat Amylase Trypsin Inhibitors Drive Intestinal Inflammation via Activation of Toll‐Like Receptor 4,” Journal of Experimental Medicine 209, no. 13 (2012): 2395–2408. 23209313 10.1084/jem.20102660 PMC3526354

[43] S. Flohe , “A Wheat‐based, Diabetes‐promoting Diet Induces a Th1‐type Cytokine Bias in the Gut of NOD Mice,” Cytokine 21, no. 3 (2003): 149–154. 12697153 10.1016/s1043-4666(02)00486-6

[44] J. C. Antvorskov , P. Fundova , K. Buschard , and D. P. Funda , “Impact of Dietary Gluten on Regulatory T Cells and Th17 Cells in BALB/c Mice,” PLoS ONE 7, no. 3 (2012): e33315. 22428018 10.1371/journal.pone.0033315 PMC3302844

[45] J. Larsen , M. Dall , J. C. Antvorskov , et al., “Dietary Gluten Increases Natural Killer Cell Cytotoxicity and Cytokine Secretion,” European Journal of Immunology 44, no. 10 (2014): 3056–3067. 25043259 10.1002/eji.201344264

[46] J. F. Bach , “The Hygiene Hypothesis in Autoimmunity: The Role of Pathogens and Commensals,” Nature Reviews Immunology 18, no. 2 (2018): 105–120. 10.1038/nri.2017.111 29034905

[47] J. F. Bach and L. Chatenoud , “The Hygiene Hypothesis: An Explanation for the Increased Frequency of Insulin‐dependent Diabetes,” Cold Spring Harbor perspectives in medicine 2, no. 2 (2012): a007799. 22355800 10.1101/cshperspect.a007799 PMC3281594

[48] V. Niederlova , “An Imbalance of Naïve and Effector T‐cell Phenotypes in Early Type 1 Diabetes Across Conventional and Regulatory Subsets,” BioRxiv (2024): 2024.

[49] Q. Li , B. Xu , S. A. Michie , K. H. Rubins , R. D. Schreriber , and H. O. Mcdevitt , “Interferon‐alpha Initiates Type 1 Diabetes in Nonobese Diabetic Mice,” PNAS 105, no. 34 (2008): 12439–12444. 18716002 10.1073/pnas.0806439105 PMC2527930

[50] C. Schuster , F. Jonas , F. Zhao , and S. Kissler , “Peripherally Induced Regulatory T Cells Contribute to the Control of Autoimmune Diabetes in the NOD Mouse Model,” European Journal of Immunology 48, no. 7 (2018): 1211–1216. 29604048 10.1002/eji.201847498 PMC6033626

[51] Y.i‐G. Chen , C.‐M. Choisy‐Rossi , T. M. Holl , et al., “Activated NKT Cells Inhibit Autoimmune Diabetes Through Tolerogenic Recruitment of Dendritic Cells to Pancreatic Lymph Nodes,” Journal of Immunology 174, no. 3 (2005): 1196–1204. 10.4049/jimmunol.174.3.1196 15661873

[52] C. M. Hull , M. Peakman , and T. I. M. Tree , “Regulatory T Cell Dysfunction in Type 1 Diabetes: What's Broken and How Can We Fix It?,” Diabetologia 60, no. 10 (2017): 1839–1850. 28770318 10.1007/s00125-017-4377-1 PMC6448885

[53] M. Feuerer , Y. Shen , D. R. Littman , C. Benoist , and D. Mathis , “How Punctual Ablation of Regulatory T Cells Unleashes an Autoimmune Lesion Within the Pancreatic Islets,” Immunity 31, no. 4 (2009): 654–664. 19818653 10.1016/j.immuni.2009.08.023 PMC2998796

[54] M. Kornete , E. Sgouroudis , and C. A. Piccirillo , “ICOS‐dependent Homeostasis and Function of Foxp3+ Regulatory T Cells in Islets of Nonobese Diabetic Mice,” Journal of Immunology 188, no. 3 (2012): 1064–1074. 10.4049/jimmunol.1101303 22227569

[55] M. Kornete , E. Mason , R. Istomine , and C. A. Piccirillo , “KLRG1 expression Identifies Short‐lived Foxp3(+) T(reg) Effector Cells With Functional Plasticity in Islets of NOD Mice,” Autoimmunity 50, no. 6 (2017): 354–362. 28850267 10.1080/08916934.2017.1364368

[56] M. Kornete , E. S. Mason , J. Girouard , E. I. Lafferty , S. Qureshi , and C. A. Piccirillo , “Th1‐Like ICOS+ Foxp3+ Treg Cells Preferentially Express CXCR3 and Home to Beta‐Islets During Pre‐Diabetes in BDC2.5 NOD Mice,” PLoS ONE 10, no. 5 (2015): e0126311. 25946021 10.1371/journal.pone.0126311 PMC4422433

[57] C. J. Dwyer , A. L. Bayer , C. Fotino , et al., “Altered Homeostasis and Development of Regulatory T Cell Subsets Represent an IL‐2R–dependent Risk for Diabetes in NOD Mice,” Science Signaling 10, no. 510 (2017): eaam9563. 29259102 10.1126/scisignal.aam9563 PMC5903848

[58] N. C. Ward , J. B. Lui , R. Hernandez , et al., “Persistent IL‐2 Receptor Signaling by IL‐2/CD25 Fusion Protein Controls Diabetes in NOD Mice by Multiple Mechanisms,” Diabetes 69, no. 11 (2020): 2400–2413. 32843568 10.2337/db20-0186 PMC7576568

[59] N. Feng , P. Vegh , E. V. Rothenberg , and M. A. Yui , “Lineage Divergence at the First TCR‐dependent Checkpoint: Preferential Gammadelta and Impaired Alphabeta T Cell Development in Nonobese Diabetic Mice,” Journal of Immunology 186, no. 2 (2011): 826–837. 10.4049/jimmunol.1002630 PMC4087166 21148803

[60] D. Funda , J. Peter Stenvang , and K. Buschard , “Age‐related Changes in T Gamma Delta Cells of NOD Mice,” Immunology Letters 45, no. 3 (1995): 179–184. 7558171 10.1016/0165-2478(95)00003-n

[61] L. C. Harrison , M. Dempsey‐Collier , D. R. Kramer , and K. Takahashi , “Aerosol Insulin Induces Regulatory CD8 Gamma Delta T Cells That Prevent Murine Insulin‐dependent Diabetes,” Journal of Experimental Medicine 184, no. 6 (1996): 2167–2174. 8976172 10.1084/jem.184.6.2167 PMC2196363

[62] N. R. Locke , S. Stankovic , D. P. Funda , and L. C. Harrison , “TCR Gamma Delta Intraepithelial Lymphocytes Are Required for Self‐tolerance,” Journal of Immunology 176, no. 11 (2006): 6553–6559. 10.4049/jimmunol.176.11.6553 16709812

[63] D. P. Funda , P. Fundova , A. K. Hansen , and K. Buschard , “Prevention or Early Cure of Type 1 Diabetes by Intranasal Administration of gliadin in NOD Mice,” PLoS ONE 9, no. 4 (2014): e94530. 24728138 10.1371/journal.pone.0094530 PMC3984166

[64] N. Duarte , M. Stenström , S. Campino , et al., “Prevention of Diabetes in Nonobese Diabetic Mice Mediated by CD1d‐restricted Nonclassical NKT Cells,” Journal of Immunology 173, no. 5 (2004): 3112–3118. 10.4049/jimmunol.173.5.3112 15322171

[65] M. Falcone , F. Facciotti , N. Ghidoli , et al., “Up‐regulation of CD1d Expression Restores the Immunoregulatory Function of NKT Cells and Prevents Autoimmune Diabetes in Nonobese Diabetic Mice,” Journal of Immunology 172, no. 10 (2004): 5908–5916. 10.4049/jimmunol.172.10.5908 15128771

[66] K. Ogasawara , J. A. Hamerman , L. R. Ehrlich , et al., “NKG2D Blockade Prevents Autoimmune Diabetes in NOD Mice,” Immunity 20, no. 6 (2004): 757–767. 15189740 10.1016/j.immuni.2004.05.008

[67] T. L. Van Belle , E. Ling , C. Haase , D. Bresson , B. Ursø , and M. G. Von Herrath , “NKG2D blockade Facilitates Diabetes Prevention by Antigen‐specific Tregs in a Virus‐induced Model of Diabetes,” Journal of Autoimmunity 40 (2013): 66–73. 22944096 10.1016/j.jaut.2012.08.001

[68] G. X. Y. Zheng , J. M. Terry , P. Belgrader , et al., “Massively Parallel Digital Transcriptional Profiling of Single Cells,” Nature Communications 8 (2017): 14049. 10.1038/ncomms14049 PMC5241818 28091601

[69] F. J. Martin , M. R. Amode , A. Aneja , et al., “Ensembl 2023,” Nucleic Acids Res. 51, no. D1 (2023): D933–D941. 36318249 10.1093/nar/gkac958 PMC9825606

[70] M. P. Lefranc , “IMGT, the International ImMunoGeneTics Information System,” Cold Spring Harbor Protocols 2011, no. 6 (2011): pdb.top115. 10.1101/pdb.top115 21632786

[71] D. Aran , A. P. Looney , L. Liu , et al., “Reference‐based Analysis of Lung Single‐cell Sequencing Reveals a Transitional Profibrotic Macrophage,” Nature Immunology 20, no. 2 (2019): 163–172. 30643263 10.1038/s41590-018-0276-y PMC6340744

[72] Y. Hao , S. Hao , E. Andersen‐Nissen , et al., “Integrated Analysis of Multimodal Single‐cell Data,” Cell 184, no. 13 (2021): 3573–3587.e29. 34062119 10.1016/j.cell.2021.04.048 PMC8238499

[73] D. Haribhai , W. Lin , B. Edwards , et al., “A central Role for Induced Regulatory T Cells in Tolerance Induction in Experimental Colitis,” Journal of Immunology 182, no. 6 (2009): 3461–3468. 10.4049/jimmunol.0802535 PMC2763205 19265124

[74] M. G. Constantinides , D. Picard , A. K. Savage , and A. Bendelac , “A Naive‐Like Population of human CD1d‐restricted T Cells Expressing Intermediate Levels of Promyelocytic Leukemia Zinc Finger,” Journal of Immunology 187, no. 1 (2011): 309–315. 10.4049/jimmunol.1100761 PMC3119760 21632718

[75] C. J. Luckey , D. Bhattacharya , A. W. Goldrath , I. L. Weissman , C. Benoist , and D. Mathis , “Memory T and Memory B Cells Share a Transcriptional Program of Self‐renewal With Long‐term Hematopoietic Stem Cells,” PNAS 103, no. 9 (2006): 3304–3309. 16492737 10.1073/pnas.0511137103 PMC1413911

