# Supplementary material for: Gluten‐Free Diet Induces Small‐Scale Changes Across Multiple T‐Cell Subsets in NOD Mice
Source: Eur J Immunol. 2025 Apr 21;55(4):e202451559. doi: 10.1002/eji.202451559 (PMC12011074; doi:10.1002/eji.202451559)
Supplement: Supplementary file 1 — Supporting information [file EJI-55-e202451559-s003.pdf]

Supplemental Table Legends

Supplemental Table 1.

Markers of the 12 main clusters related to Figure 1C.

Supplemental Table 2.

Differentially expressed genes between mice fed with STD and GFD related to Figure 5A.

Supplemental Figures

Fig S1

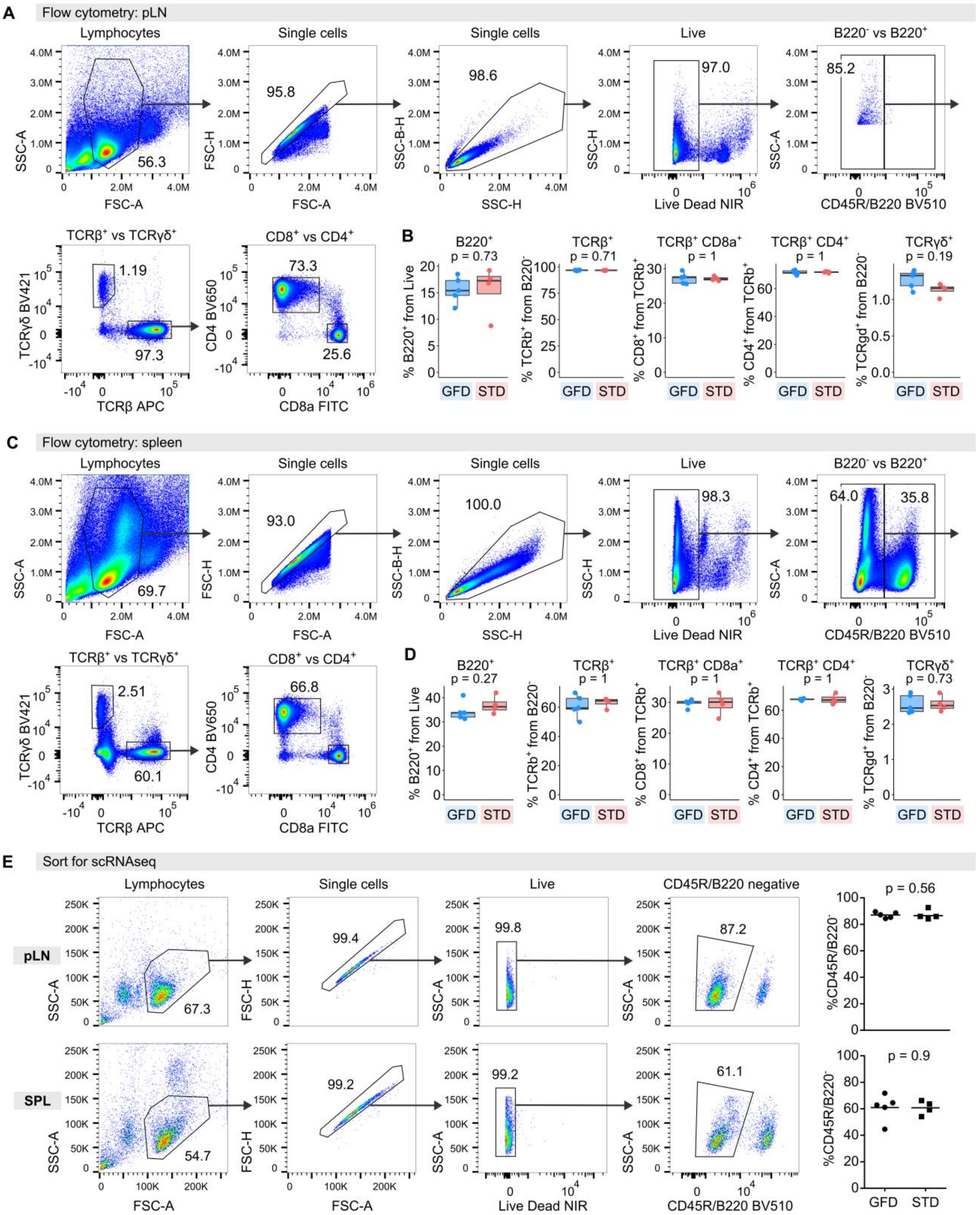

Supplemental Figure 1. Flow cytometry analysis of GF vs STD mice.

Female NOD mice were housed on STD or GFD and analyzed by flow cytometry at the age of 6 weeks. **(A-D)** Flow cytometry analysis of B cells, T cells and T-cell populations in the cohort of 6 weeks old NOD mice on the STD and GFD that was analyzed by scRNAseq. **(A)** Representative gating of populations in pancreatic lymph nodes (pLN). **(B)** Quantification of cell populations shown in (A). **(C)** Representative gating of populations in spleen. **(D)** Quantification of cell populations shown in (C). **(E)** Representative gating strategy for flow cytometry sorting of B220- cells used for scRNAseq profiling. On right, quantification of the abundance of non-B cells that were used for scRNAseq analysis. Line at median. In the boxplots, hinges correspond to the first and third quartiles, the whiskers represent 1.5x inter-quartile range. P-values were determined by two-tailed Mann-Whitney test. Data from one experiment, n = 5 for mice on GFD and n = 4 for mice on STD. GFD – gluten-free diet, STD – standard diet, pLN – pancreatic lymph nodes, SPL – spleen.

Fig S2

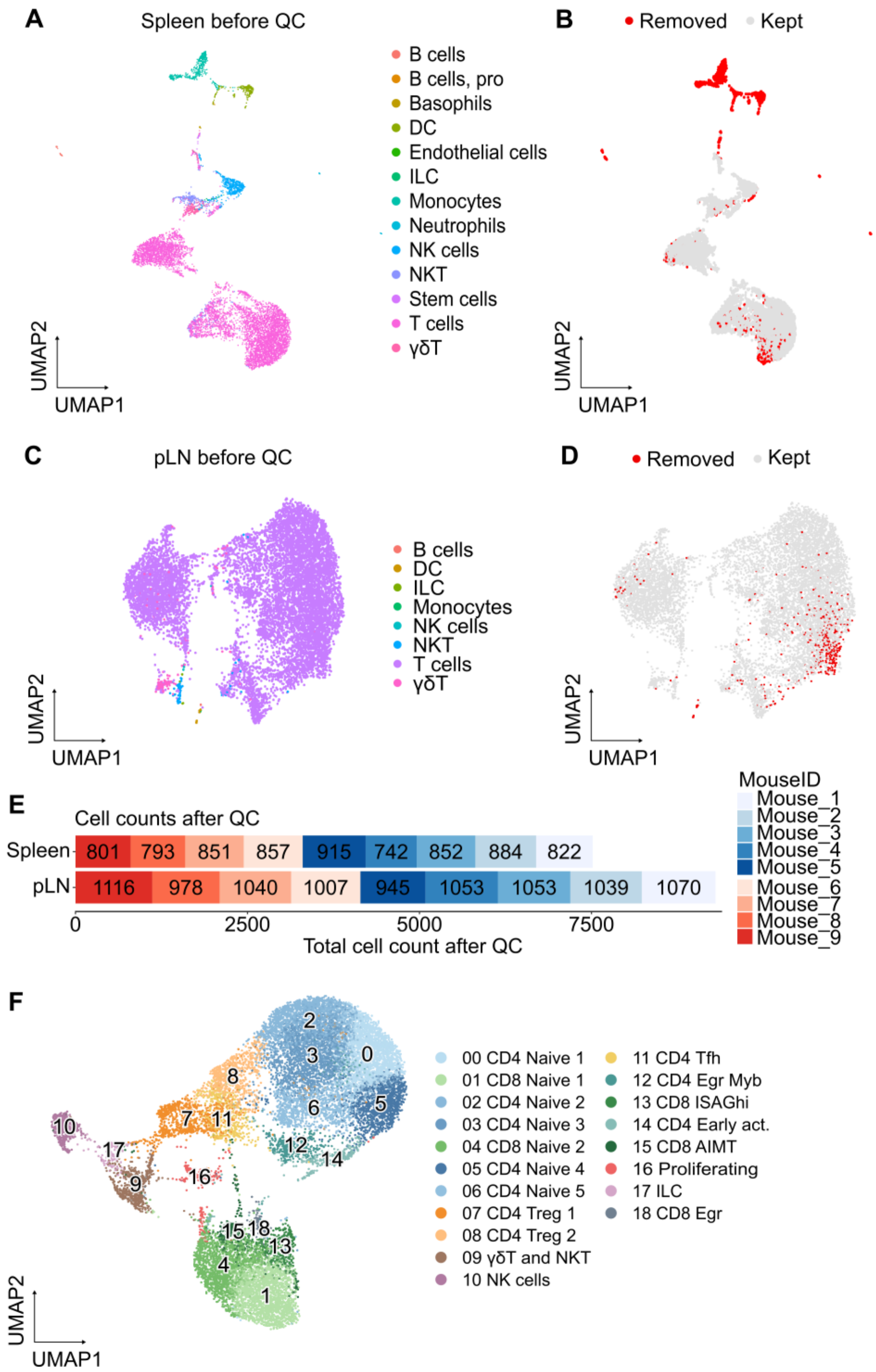
A
Spleen before QC
B cells
B cells, pro
Basophils
DC
Endothelial cells
ILC
Monocytes
Neutrophils
NK cells
NKT
Stem cells
T cells
γδT
UMAP2
UMAP1
B
Removed
Kept
UMAP2
UMAP1
C
pLN before QC
B cells
DC
ILC
Monocytes
NK cells
NKT
T cells
γδT
UMAP2
UMAP1
D
Removed
Kept
UMAP2
UMAP1
E
Cell counts after QC
Spleen
801 793 851 857 915 742 852 884 822
pLN
1116 978 1040 1007 945 1053 1053 1039 1070
0
2500
5000
7500
Total cell count after QC
MouseID
Mouse_1
Mouse_2
Mouse_3
Mouse_4
Mouse_5
Mouse_6
Mouse_7
Mouse_8
Mouse_9
F
00 CD4 Naive 1
01 CD8 Naive 1
02 CD4 Naive 2
03 CD4 Naive 3
04 CD8 Naive 2
05 CD4 Naive 4
06 CD4 Naive 5
07 CD4 Treg 1
08 CD4 Treg 2
09 γδT and NKT
10 NK cells
11 CD4 Tfh
12 CD4 Egr Myb
13 CD8 ISAGhi
14 CD4 Early act.
15 CD8 AIMT
16 Proliferating
17 ILC
18 CD8 Egr
UMAP2
UMAP1

Supplemental Figure 2. Quality control of scRNAseq data and validation by flow cytometry.

Female NOD mice were housed on STD or GFD and analyzed by scRNAseq at the age of 6 weeks. **(A)** Cells from spleen were analyzed by scRNAseq and automatically annotated with Immgen immune dataset annotations. n = 8827 cells from 9 mice in one experiment. **(B)** The same UMAP projection as in (A). Cells with other than T-lymphocyte profile and cells with low quality estimated by more than 7.5% of mitochondrial genes or fewer than 750 detected UMI were removed. **(C)** Cells from pLN were analyzed by scRNAseq and automatically annotated with Immgen immune dataset annotations. n = 9742 cells from 9 mice in one experiment. **(D)** The same UMAP projection as in (C). Cells with other than T-lymphocyte profile and cells with low quality estimated by more than 7.5% of mitochondrial genes or fewer than 750 detected UMI were removed. **(E)** Counts of cells retained after quality control shown in (B) and (D) in the spleen and pLN datasets, respectively. Numbers represent counts of cells per each mouse analyzed. Red color indicates standard diet, blue color gluten-free diet. **(F)** The same UMAP projection of scRNAseq data as in Fig. 1D. Unsupervised clustering of the merged spleen and pLN datasets showing 19 populations with manual annotations. pLN – pancreatic lymph nodes, QC – quality control.

Fig S3

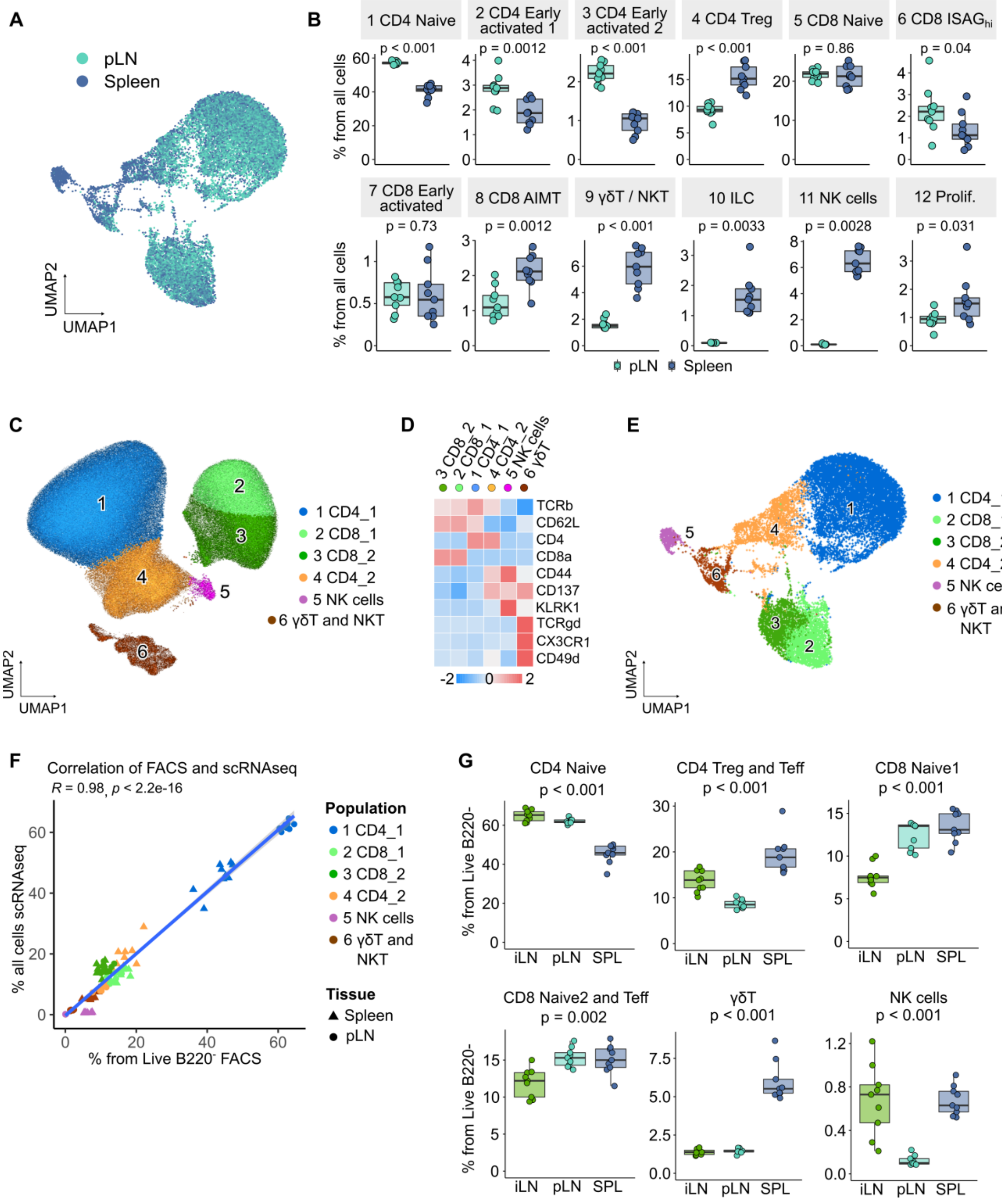
A
pLN
Spleen
UMAP2
UMAP1
B
1 CD4 Naive
2 CD4 Early activated 1
3 CD4 Early activated 2
4 CD4 Treg
5 CD8 Naive
6 CD8 ISAG$_{hi}$
p < 0.001
p = 0.0012
p < 0.001
p < 0.001
p = 0.86
p = 0.04
% from all cells
7 CD8 Early activated
8 CD8 AIMT
9 γδT / NKT
10 ILC
11 NK cells
12 Prolif.
p = 0.73
p = 0.0012
p < 0.001
p = 0.0033
p = 0.0028
p = 0.031
pLN Spleen
C
1 CD4_1
2 CD8_1
3 CD8_2
4 CD4_2
5 NK cells
6 γδT and NKT
D
3 CD8_2
2 CD8_1
1 CD4_1
4 CD4_2
5 NK cells
6 γδT
TCRb
CD62L
CD4
CD8a
CD44
CD137
KLRK1
TCRgd
CX3CR1
CD49d
E
1 CD4_1
2 CD8_1
3 CD8_2
4 CD4_2
5 NK cells
6 γδT and NKT
F
Correlation of FACS and scRNAseq
R = 0.98, p < 2.2e-16
% all cells scRNAseq
% from Live B220- FACS
Population
1 CD4_1
2 CD8_1
3 CD8_2
4 CD4_2
5 NK cells
6 γδT and NKT
Tissue
Spleen
pLN
G
CD4 Naive
p < 0.001
CD4 Treg and Teff
p < 0.001
CD8 Naive1
p < 0.001
% from Live B220-
iLN pLN SPL
CD8 Naive2 and Teff
p = 0.002
γδT
p < 0.001
NK cells
p < 0.001

Supplemental Figure 3. ScRNAseq and flow cytometry analysis of selected T-cell subsets.

Female NOD mice were housed on STD or GFD and analyzed by scRNAseq and flow cytometry at the age of 6 weeks. **(A)** The same UMAP projection as in Fig 1D. Cells are colored by source tissue. pLN – pancreatic lymph node. **(B)** Quantification of abundance of clusters shown in (Fig 1D) in organs represented in (A). Plotted are percentages of cells in the specified cluster from all cells. P-values were determined by two-tailed Mann-Whitney test. In the boxplots, the line represents median, the hinges correspond to the first and third quartiles, the whiskers represent 1.5x inter-quartile range. **(C)** High dimensional analysis of flow cytometry data from the same cohort of mice that was profiled by scRNAseq (n = 9 mice). Cells are colored by manual annotations. **(D)** Heatmap of marker proteins that characterize cell populations presented in (C) detected by flow cytometry. Color represents column-scaled z-score of average fluorescence intensity of a protein in a cell population. **(E)** The same UMAP projection of scRNAseq data as in Fig. 1D. Selected clusters were merged to represent the same populations as in flow cytometry analysis. Cells are colored by manual annotations. **(F)** Correlation of percentages of populations detected by flow cytometry (showed in C) and scRNAseq analysis (shown in E). P-value was determined by the Spearman's correlation test. **(G)** Quantification of abundance of cell populations shown in (C) in the three organs analyzed by flow cytometry. Plotted are percentages of cells in the specified cluster from gated Live B220- cells. P-values were determined by Kruskal-Wallis test. In the boxplots, the line represents median, the hinges correspond to the first and third quartiles, the whiskers represent 1.5x inter-quartile range. iLN – inguinal lymph nodes, pLN – pancreatic lymph nodes, SPL – spleen.

Fig S4

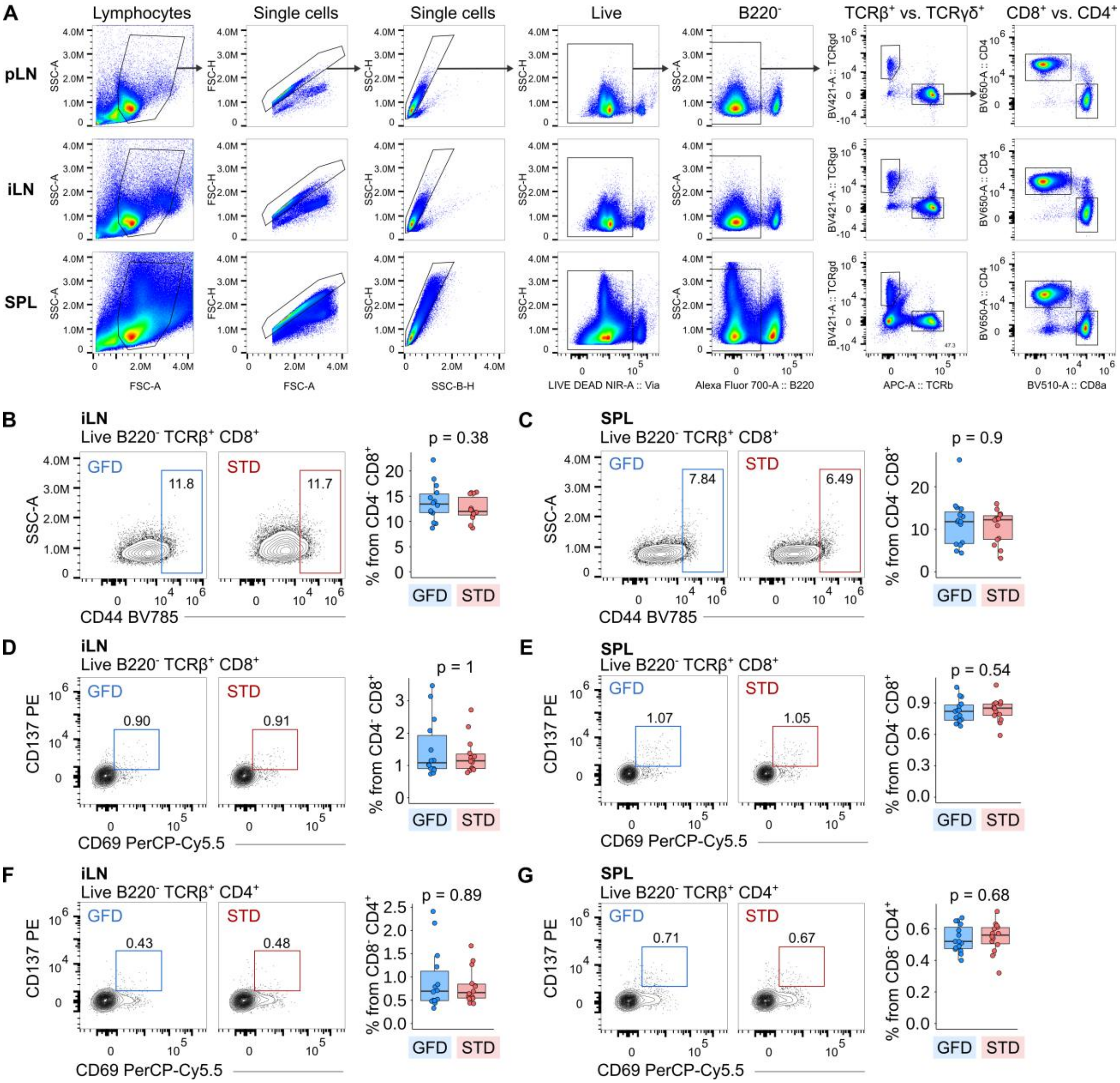
A
Lymphocytes
Single cells
Single cells
Live
B220⁻
TCRβ⁺ vs. TCRγδ⁺
CD8⁺ vs. CD4⁺
pLN
iLN
SPL
SSC-A
FSC-A
FSC-H
SSC-H
SSC-B-H
LIVE DEAD NIR-A :: Via
Alexa Fluor 700-A :: B220
BV421-A :: TCRgd
APC-A :: TCRb
BV650-A :: CD4
BV510-A :: CD8a
47.3
B
iLN
Live B220⁻ TCRβ⁺ CD8⁺
GFD
STD
11.8
11.7
p = 0.38
% from CD4⁻ CD8⁺
CD44 BV785
C
SPL
Live B220⁻ TCRβ⁺ CD8⁺
7.84
6.49
p = 0.9
D
iLN
Live B220⁻ TCRβ⁺ CD8⁺
CD137 PE
0.90
0.91
p = 1
CD69 PerCP-Cy5.5
E
SPL
Live B220⁻ TCRβ⁺ CD8⁺
1.07
1.05
p = 0.54
F
iLN
Live B220⁻ TCRβ⁺ CD4⁺
0.43
0.48
p = 0.89
% from CD8⁻ CD4⁺
G
SPL
Live B220⁻ TCRβ⁺ CD4⁺
0.71
0.67
p = 0.68

Supplemental Figure 4. Flow cytometry analysis of selected T-cell subsets.

Female NOD mice were housed on STD or GFD and analyzed by scRNAseq and flow cytometry at the age of 6-8 weeks. **(A)** Representative gating of populations by flow cytometry extracellular staining in pLN (upper), iLN (middle) and SPL (lower). **(B-G)** Flow cytometry analysis of selected cell subsets: $CD8^+$ $CD44^+$ cells (B, C), $CD69^+CD137^+$ $CD8^+$ T cells (D, E) and $CD69^+CD137^+$ $CD4^+$ T cells (F, G) in the iLN (B,D,F) or spleen (C,E,G) of mice fed with gluten-free (GFD, blue) or standard (STD, red) diet. Left – representative gating of the population in one mouse housed on gluten-free diet (GFD) (blue) and one mouse housed on standard diet (STD) (red). Right – quantification of the percentages of selected T-cell population from parent population in mice housed on GFD or STD. P-values were determined by two-tailed Mann-Whitney test. n = 29 mice from three independent experiments. In the boxplots, the line represents median, the hinges correspond to the first and third quartiles, the whiskers represent 1.5x inter-quartile range. GFD – gluten-free diet, STD – standard diet, iLN – inguinal lymph nodes, pLN – pancreatic lymph nodes, SPL – spleen.

Fig S5

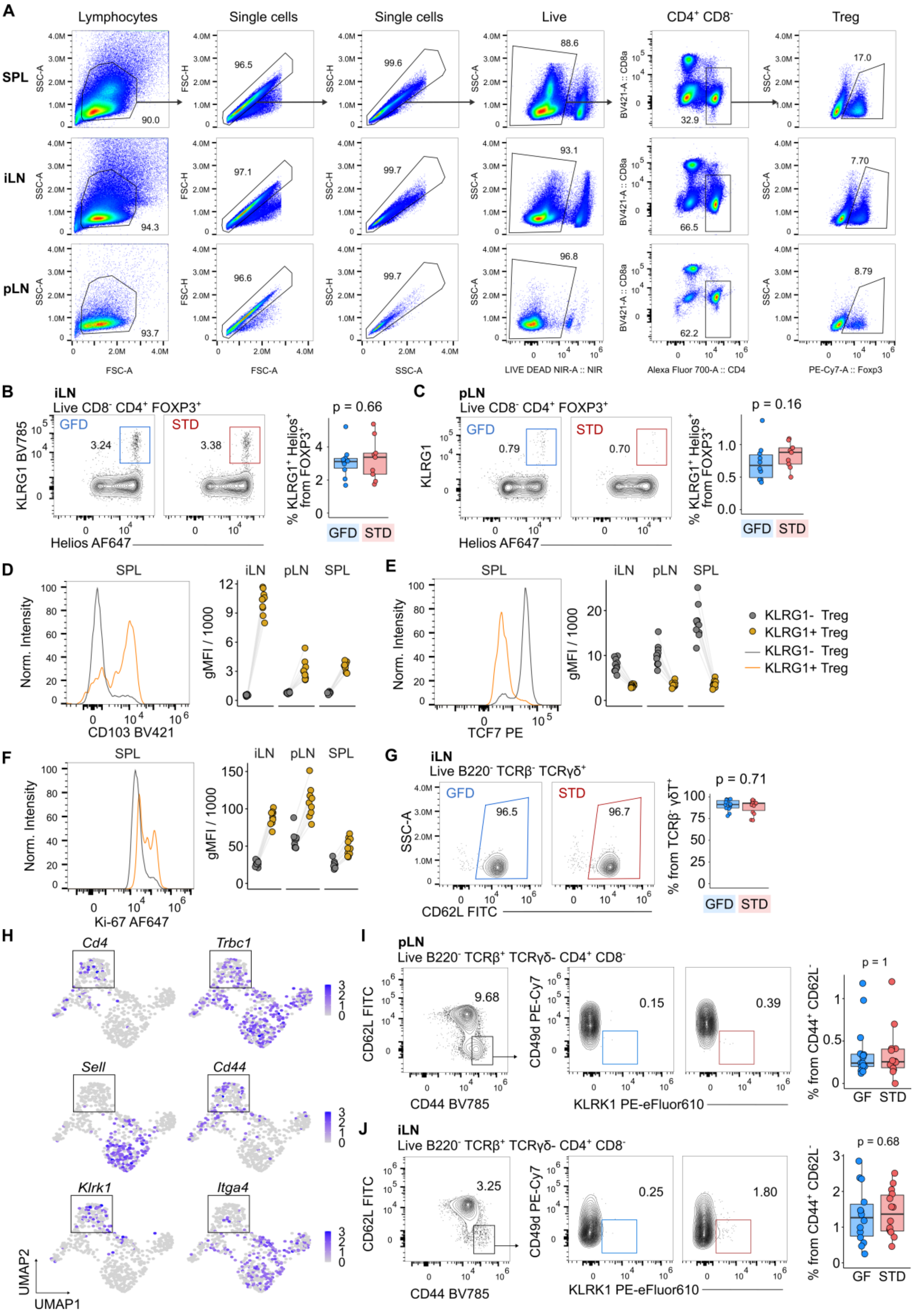
A
Lymphocytes
Single cells
Single cells
Live
CD4+ CD8-
Treg
SPL
iLN
pLN
SSC-A
FSC-H
SSC-H
BV421-A :: CD8a
FSC-A
SSC-A
LIVE DEAD NIR-A :: NIR
Alexa Fluor 700-A :: CD4
PE-Cy7-A :: Foxp3
90.0
96.5
99.6
88.6
32.9
17.0
94.3
97.1
99.7
93.1
66.5
7.70
93.7
96.6
99.7
96.8
62.2
8.79
B
iLN
Live CD8- CD4+ FOXP3+
GFD
STD
3.24
3.38
KLRG1 BV785
Helios AF647
p = 0.66
% KLRG1+ Helios+ from FOXP3+
GFD
STD
C
pLN
Live CD8- CD4+ FOXP3+
GFD
STD
0.79
0.70
KLRG1
Helios AF647
p = 0.16
% KLRG1+ Helios+ from FOXP3+
GFD
STD
D
SPL
Norm. Intensity
CD103 BV421
iLN
pLN
SPL
gMFI / 1000
E
SPL
Norm. Intensity
TCF7 PE
iLN
pLN
SPL
gMFI / 1000
KLRG1- Treg
KLRG1+ Treg
KLRG1- Treg
KLRG1+ Treg
F
SPL
Norm. Intensity
Ki-67 AF647
iLN
pLN
SPL
gMFI / 1000
G
iLN
Live B220- TCRβ- TCRγδ+
GFD
STD
96.5
96.7
SSC-A
CD62L FITC
p = 0.71
% from TCRβ- γδT+
GFD
STD
H
Cd4
Trbc1
Sell
Cd44
Klrk1
Itga4
UMAP2
UMAP1
I
pLN
Live B220- TCRβ+ TCRγδ- CD4+ CD8-
9.68
0.15
0.39
CD62L FITC
CD44 BV785
CD49d PE-Cy7
KLRK1 PE-eFluor610
p = 1
% from CD44+ CD62L-
GF
STD
J
iLN
Live B220- TCRβ+ TCRγδ- CD4+ CD8-
3.25
0.25
1.80
CD62L FITC
CD44 BV785
CD49d PE-Cy7
KLRK1 PE-eFluor610
p = 0.68
% from CD44+ CD62L-
GF
STD

Supplemental Figure 5. Flow cytometry analysis of Treg cells, Tgd cells and NKT cells.

Female NOD mice were housed on STD or GFD and analyzed by scRNAseq at the age of 6 weeks and by flow cytometry at the age of 6-8 weeks. **(A)** Representative gating of populations by flow cytometry intracellular staining in SPL (upper), iLN (middle) and pLN (lower). **(B-C)** Flow cytometry analysis of KLRG1^{+} Helios^{+} Treg. Left – representative gating of the population in an iLN (B) or pLN (C) sample from a representative mouse housed on GFD (blue) and a representative mouse housed on STD (red). Right – quantification of the percentages of KLRG1^{+} Helios^{+} Treg from all FOXP3^{+} Tregs in mice on GFD and on STD. P-values were determined by two-tailed Mann-Whitney test. n = 19 mice from two independent experiments. In the boxplots, the line represents median, the hinges correspond to the first and third quartiles, the whiskers represent 1.5x inter-quartile range. **(D-F)** Flow cytometry analysis of the intensity of CD103 (D), TCF7 (E) and KI-67 (F) in KLRG1^{+} (orange) vs. KLRG1^{-} (grey) Treg cells. Shown is the representative histogram of staining in the spleen from one animal (left) and quantification in the iLN, pLN and SPL of 10 animals. n = 10 from one experiment. **(G)** Flow cytometry analysis of manually gated TCRβ^{-} TCRγδ^{+} CD62L^{+} cells in iLN. Left – representative gating of the population in one mouse housed on GFD (blue) and one mouse housed on STD (red). Right – quantification of the percentage of TCRγδ^{+} CD62L^{+} T cells in mice on GFD and on STD. P-values were determined by two-tailed Mann-Whitney test. n = 29 mice from three independent experiments. In the boxplots, the line represents median, the hinges correspond to the first and third quartiles, the whiskers represent 1.5x inter-quartile range. **(H)** The same UMAP plots as in Fig 4A. Cells are colored by expression of selected genes which were used for flow cytometry gating. **(I-J)** Flow cytometry analysis of NKT1 cells in pLN (I) and iLN (J). Left – representative gating of CD44^{+} CD62L^{-} T cells. Middle – representative gating of CD49d^{-} KLRK^{+} cells (NKT1) in one mouse housed on GFD (blue) and one mouse housed on STD (red). Right – quantification of the percentage of NKT1 cells in mice on GFD and on STD. P-values were determined by two-tailed Mann-Whitney test. n = 29 mice from three independent experiments. In the boxplots, the line represents median, the hinges correspond to the first and third quartiles, the whiskers represent 1.5x inter-quartile range. GFD – gluten-free diet, STD – standard diet, iLN – inguinal lymph nodes, pLN – pancreatic lymph nodes, SPL – spleen.
